# Expression of DP2 (CRTh2), a Prostaglandin D_2_ Receptor, in Human Mast Cells

**DOI:** 10.1371/journal.pone.0108595

**Published:** 2014-09-30

**Authors:** Tae Chul Moon, Eduardo Campos-Alberto, Tsuyoshi Yoshimura, Graeme Bredo, Aja M. Rieger, Lakshmi Puttagunta, Daniel R. Barreda, A. Dean Befus, Lisa Cameron

**Affiliations:** 1 Pulmonary Research Group, Department of Medicine, University of Alberta, Edmonton, AB, Canada; 2 Department of Biological Sciences, University of Alberta, Edmonton, AB, Canada; 3 Department of Laboratory Medicine and Pathology, University of Alberta Hospitals, Edmonton, AB, Canada; 4 Department of Agricultural, Food and Nutritional Science, University of Alberta, Edmonton, AB, Canada; 5 Department of Pathology and Laboratory Medicine, Schulich School of Medicine & Dentistry, Western University, London, ON, Canada; Medical School of Hannover, Germany

## Abstract

PGD_2_ has long been implicated in allergic diseases. Recent cloning of a second PGD_2_ receptor, DP2 (also known as CRTh2), led to a greater understanding of the physiological and pathophysiological implications of PGD_2_. PGD_2_ signaling through DP1 and DP2 mediates different and often opposite effects in many cell types of the immune system. Although mast cells (MC) are the largest source of PGD_2_ in the body, there is little information about their potential expression of DP2 and its functional significance. In this study, we show that tissue MC in human nasal polyps express DP2 protein, and that human MC lines and primary cultured human MC express mRNA as well as protein of DP2. By immunohistochemistry, we detected that 34% of MC in human nasal polyps expressed DP2. In addition, flow cytometry showed that 87% of the LAD2 human MC line and 98% of primary cultured human MC contained intracellular DP2. However, we could not detect surface expression of DP2 on human MC by single cell analysis using imaging flow cytometry. Blocking of endogenous PGD_2_ production with aspirin did not induce surface expression of DP2 in human MC. Two DP2 selective agonists, DK-PGD_2_ and 15R-15-methyl PGD_2_ induced dose-dependent intracellular calcium mobilization that was abrogated by pertussis toxin, but not by three DP2 selective antagonists. MC mediator release including degranulation was not affected by DP2 selective agonists. Thus, human MC express DP2 intracellularly rather than on their surface, and the function of DP2 in human MC is different than in other immune cells such as Th2 cells, eosinophils and basophils where it is expressed on the cell surface and induces Th2 cytokine and/or granule associated mediator release. Further studies to elucidate the role of intracellular DP2 in human MC may expand our understanding of this molecule and provide novel therapeutic opportunities.

## Introduction

Mast cells (MC) are tissue-resident cells derived from bone marrow progenitors. They are widely distributed throughout the body, performing multiple tasks in different locations and functional settings. MC are primary effector cells of allergic inflammation following IgE cross-linking and they also have diverse roles in angiogenesis, wound healing, tissue remodeling, regulation of inflammation, host defense, and innate and adaptive immune responses [1]–[4]. Along with mediators such as histamine and proteases in the granules, and *de novo* synthesized cytokines and chemokines, activated MC produce an abundance of prostaglandin (PG) D_2_ and leukotriene (LT) C_4_ [5], [6]. These lipid mediators have bronchoconstricting and vasoactive properties, but also participate in host defense, inflammation, and allergic diseases through diverse activities such as effector cell trafficking, antigen presentation, immune cell activation and fibrosis [6]–[8].

PGD_2_ is a key mediator produced by activated MC [5], [9] and antigen presenting cells [10] following allergen exposure in patients with asthma, atopic dermatitis or allergic rhinitis [11]–[13]. PGD_2_ contributes directly to smooth muscle contraction [14], [15], vascular leak and vasodilation [16] that typically occur in type I hypersensitivity, and also potentiates cellular responses to other physiologically relevant mediators (eg., histamine) released during these allergic reactions [17]. It modulates dendritic cell migration and maturation [18] and induces migration and activation of human Th2 cells [19], [20], eosinophils [21], [22], basophils [20], [23], and macrophages [24]. PGD_2_ mediates its effects *via* activation of D prostanoid receptors (DPs). DP1, a member of the prostanoid family of G protein-coupled receptors (GPCR), uses pertussis toxin (PTX)-resistant G_s_ proteins for its signaling that stimulates adenylate cyclase and elevates intracellular levels of cyclic adenosine monophosphate (cAMP). Recently, DP1 was shown to play a role in MC maturation toward an anaphylaxis-sensitive phenotype [25]. DP2 [also known as CRTh2 (chemoattractant receptor-homologous molecule expressed on Th2 cells), GPR44, and CD294] is a GPCR of the formylmethionylleucylphenylalanine receptor subfamily with a primary amino acid sequence homology to chemokine receptors. It signals with PTX-sensitive G_i_ proteins that suppress adenylate cyclase and decrease intracellular cAMP levels, but induces intracellular Ca^2+^ mobilization in response to PGD_2_ [20], [26], [27]. Although DP2 was first discovered in human Th2 cells and is a specific marker for human Th2 compared to human Th1 cells, this differs in the mouse where both Th1 and Th2 cells express DP2 [28]. Human and/or mouse eosinophils, basophils, macrophages and dendritic cells express DP2, and DP2 signaling causes chemotaxis and activation of these cells [18]–[24], [26], [29], [30].

Although MC are a major source of PGD_2_, little is known about DP2 expression in human MC except for an immunohistochemical study which shows DP2 expression in human nasal mucosa MC [30]. In mouse, DP2 transcripts have been identified in MC lines (P815, MC/9) [28] and bone-marrow derived primary cultured MC [31]. Boehme *et al* reported that DP2 in murine bone marrow-derived MC is involved in chemotaxis, down-regulation of CD62L, and up-regulation of CD23 and CD30 [31]. However, given differences between human and mouse in structure of the DP2 gene [32] and in expression of DP2 in Th2 and Th1 cells, the functions of DP2 in human and mouse MC might differ. Thus, we examined for the first time whether DP2 is expressed on human MC and if ligation of DP2 influences human MC activation.

## Materials and Methods

### Cell culture

HMC-1 (human mast cell line-1), an immature MC line derived from a patient with MC leukemia (a gift from Dr. J.H. Butterfield, Rochester, MN) [33], and LAD2 (laboratory of allergic diseases 2), developed from human bone marrow mononuclear cells (generously provided by Drs. D.D. Metcalfe and A. Kirshenbaum, National Institutes of Health, Bethesda, MD) [34] were cultured as previously described [35].

Human peripheral blood-derived primary cultured MC (hPBDMC) and cord blood-derived primary cultured MC (hCBDMC) were developed from CD34^+^ progenitors as previously described with minor modifications [35]–[38]. Briefly, approximately 100 mL of peripheral blood drawn from healthy donors into 10 mL heparinized Vacutainer tubes (BD Canada, Oakville, ON, Canada), or EDTA-treated umbilical cord blood from placentae obtained within 45 min of delivery were used. Ethics about the studies using human peripheral blood from healthy donor and cord blood from placentae after delivery were approved by the Human Ethics Research Committee, University of Alberta and Capital Health Region, and written informed consent for the use of donated peripheral blood or cord blood from placentae was obtained from each donor. The blood was diluted with the same volume of 10 mM phosphate buffer (pH 7.4) containing 150 mM NaCl [phosphate-buffered saline (PBS)] and then layered on Histopaque 1077 (Sigma-Aldrich Canada Ltd., Oakville, ON, Canada). The mononuclear cell fraction was obtained after centrifugation at 450×g for 30 min. After washing the mononuclear cells twice with PBS, CD34^+^ progenitors were isolated using the EasySep human CD34 positive selection kit (StemCell Technologies, Vancouver, BC, Canada). CD34^+^ cells from peripheral blood were cultured at 5×10^4^ cells/mL in StemSpan SFEM (StemCell Technologies) supplemented with 100 ng/mL rhSCF (PeproTech Inc., Rocky Hill, NJ) and 100 ng/mL rhIL-6 (PeproTech Inc.) for 8 wk, with 30 ng/mL rhIL-3 (PeproTech Inc.) used for the first wk only for hPBDMC cultures. CD34^+^ cells from cord blood were cultured in AIM-V (Life Technologies) supplemented with 100 ng/mL rhSCF for 8 wk to develop hCBDMC. The StemSpan SFEM or AIM-V was hemidepleted twice a wk. At 4 wk, the entire volume of old media was replaced once by fresh media and then hemidepleted twice a wk until 8 wk. Primary MC cultures were used after 8 wk and confirmed as> 99% MC by tryptase/chymase staining before use [38], [39].

The DP2-transfected line K562/B19 and its control line K562/neo was generously provided by Dr. K. Nagata (BML, Inc., Saitama, Japan) and cultured as previously described [40].

### Reverse transcription-polymerase chain reaction (RT-PCR)

Total RNA was extracted with RNAqueous-4PCR kit (Life Technologies) according to manufacturer's instructions and quantified by measuring optical density at 260 nm. Purity and integrity of extracted RNA were assessed by 260/280 nm ratio and applying to 1.2% formaldehyde-agarose gels, respectively. RT-PCR was carried out using SuperScript III First-Strand Synthesis System for RT-PCR (Life Technologies). Five µg of total RNA from each sample was used as template for reverse transcription reaction. The RNA/primer mixture (5 µg total RNA, 5 µM oligo(dT)_20_ primers and 1 mM dNTP mixture in 10 µL DEPC-treated water) was incubated for 5 min at 65°C, then placed on ice for at least 1 min. Ten µL cDNA synthesis mixture (200 U SuperScript III reverse transcriptase, 40 units RNaseOUT, 20 mM DTT, 10 mM MgCl_2_, in 2× RT buffer) was added and incubated for 50 min at 50°C, 5 min at 85°C and chilled on ice. Before proceeding to PCR, 2 U of RNase H were added and incubated for 20 min at 37°C. Two µL of the above cDNA was used for PCR with 1 U Red Taq DNA Polymerase (Sigma). PCR was carried out with Mastercycler Gradient (Eppendorf, Mississauga, ON, Canada). The specific primers were designed based on published sequence data: human DP2 (301 bp) forward 5′-CCT CTG TGC CCA GAG CCC CAC GAT GTC GGC-3′, reverse 5′-CAC GGC CAA GAA GTA GGT GAA GAA G-3′ [41]; DP1 (635 bp) forward 5'-CTT CTA CCG ACG GCA CAT CAC C-3', reverse 5'-TGC ACC GGC TCC TGT ACC TAA G-3' [42]; β-actin (326 bp) forward 5'-GGC ATC CTC ACC CTG AAG TA-3', reverse 5'-AGG GCA TAC CCC TCG TAG AT-3' [43]. We optimized the cycle number to be within the exponential phase of amplification. PCR with 2 µL of DNase/RNase free water (Sigma) instead of cDNA was run as a negative control and cDNA from human Th2 cells (CRTh2^+^/CD4^+^ T cells differentiated *in vitro* by culturing in Th2-polarizing conditions [44]) was used as a positive control for DP2. The PCR products were analyzed by 1% agarose gel electrophoresis with ethidium bromide staining and confirmed by sequencing (DNA Core Services Lab, University of Alberta).

### Immunostaining of mast cells

To study potential surface expression of DP2, 2.5×10^5^ cells in culture media were washed with PBS-FACS buffer (1× PBS containing 0.5% BSA, 0.1% NaN_3_ and 3% FBS) then resuspended with 100 µL PBS-FACS buffer. After blocking FcR with 1 µL human FcR blocking reagent (Miltenyi Biotec, Auburn, CA) and 50.1 µg normal mouse IgG (Life Technologies) for 30 min at room temperature (RT), cells were incubated with specific Ab and isotype matched control Ab directly conjugated with fluorophore at 4°C for 30 min: APC-conjugated mouse anti-human DP2 IgG_2A_ (R&D Systems Inc., Minneapolis, MN) and APC-conjugated mouse IgG_2A_ (R&D Systems Inc.); FITC-conjugated mouse anti-human FcεRIα IgG_2b_ (eBioscience, San Diego, CA) and FITC-conjugated mouse IgG_2b_ (eBioscience). Stained cells were washed with 1 mL PBS-FACS buffer, fixed with 200 µL PBS-FACS containing 2% paraformaldehyde and 0.54% sucrose, and fluorescence read using a FACSCalibur (BD Biosciences, Mississauga, ON, Canada), FACSCanto II (BD Biosciences) or an ImageStream Mark II (Amnis Co., EMD Millipore, Seattle, WA) [45]. For ImageStream Mark II analyses, nuclei were also stained with 30 µM 4',6-diamidino-2-phenylindole (DAPI, Life Technologies).

For total expression (surface and intracellular) of DP2, cells were fixed with 100 µL 4% paraformaldehyde for 10 min on ice, permeabilized using 0.4% saponin for 10 min on ice, and FcR were blocked with 1 µL human FcR blocking reagent (Miltenyi Biotec) and 50.1 µg normal mouse IgG for 30 min at RT before staining with Ab. Data were analyzed with WinMDI ver. 2.9 (developed by Joe Trotter), FlowJo ver. 10.0.5 (Tree Star, Inc., Ashland, OR) or IDEAS software (Amnis).

### Immunohistochemical staining of human nasal polyp mast cells

Nasal polyps were obtained from endoscopic sinus surgery from patients with chronic rhinosinusitis at the University of Alberta Hospital, Canada, from archives of 2007 to 2009 (*n* = 15) [39]. All studies were approved by the Human Ethics Research Committee, University of Alberta. Written informed consent for the use of tissues was obtained from each patient by a surgical release form signed before surgery, explaining that any tissue removed from the patient may be used for diagnosis, research, or disposal. After excision, tissue samples were placed in 10% neutral buffered formalin and then 4 µm sections were generated from each tissue block after dehydration and paraffin embedding. After heat-induced epitope retrieval (20 min at 90–95°C) using Target Retrieval Solution (Citrate pH 6.0, Dako, Burlington, ON, Canada), deparaffinized sections were incubated with 4% hydrogen peroxide in methanol for 20 min to reduce endogenous peroxidase activity. Sections were incubated in blocking solution (5% normal goat serum in PBS) for 30 min before incubation in primary Ab [rabbit anti-human DP2 IgG (Abcam Inc., Toronto, ON, Canada) and alkaline phosphatase–conjugated mouse anti-human MC tryptase (G3) IgG_1_ (EMD Millipore, Billerica, MA)] or isotype matched control Ab [normal rabbit IgG (AbD Serotec, Raleigh, NC) and alkaline phosphatase–conjugated mouse IgG (Abcam Inc.)] overnight at 4°C. Sections were washed 3 times with PBS, incubated for 30 min at RT with biotin-conjugated goat anti-rabbit IgG (Vector Laboratories Inc., Burlingame, CA), washed 3 times with PBS, and incubated for 1 h at RT with horseradish peroxidase (HRP)-conjugated streptavidin (Vector Laboratories Inc.) for DP2 staining. The sections were developed using the NovaRED peroxidase substrate kit (Vector Laboratories Inc.) and alkaline phosphatase substrate kit III (Vector Laboratories Inc.), respectively. Coverslips were placed on the slides with mounting medium (Cytoseal-XYL, Richard-Allan Scientific, Kalamazoo, MI). For morphometric analyses of the abundance of DP2 positive cells and of MC, three high-powered fields (HPF) distant from the edge of the section on each slide were randomly selected, and either single or double positive cells were counted using a microscope (magnification 10×40, HPF = 0.196 mm^2^). Total cell numbers (excluding epithelial cells) in a field were determined by counting nuclei. Photography was taken using DXM1200C digital camera (Nikon Canada Inc., Mississauga, ON, Canada) attached to Eclipse E600W microscope (Nikon Canada Inc.).

### Intracellular calcium (Ca^2+^) flux

Intracellular Ca^2+^ flux was measured using Fluo-4 NW Calcium Assay kit (Life Technologies) according to manufacturer's instructions. After measuring baseline fluorescence of Fluo-4 AM loaded MC (1.25×10^5^ cells in 50 µL/well) for 100 sec, 100 nM to 10 µM of DP2 agonist [PGD_2_ (Cayman Chemical, Ann Arbor, MI), 15R-15-methyl PGD_2_ (Cayman Chemical), or 13,14-dihydro-15-keto PGD_2_ (Cayman Chemical)] was added and intracellular Ca^2+^ response was measured using fluorescence plate reader (FLx800, Bio-Tek Instruments Inc., Winooski, VT) with excitation and emission wavelengths of 485 nm and 516 nm, respectively. To antagonize DP2, DP2 selective antagonists [1 µM CAY10471 (Cayman Chemical) or 100 nM CAY10595 (Cayman Chemical)] or DP2/TP dual antagonist [1 µM ramatroban (Cayman Chemical)] was added 5 min before agonist treatment. Cells were pretreated with 10 nM PTX for 2 h to inhibit Gα_i_ before loading Fluo-4 AM. Cytosolic free Ca^2+^ was presented by one of the following calculations: Fluorescence ratio (fluorescence unit at each time point/baseline fluorescence unit), Δfluorescence ratio (fluorescence ratio of agonist treated MC – fluorescence ratio of sham treated MC), integral (area under the curve during indicated time period) or Δintegral (integral of agonist treated MC – integral of sham treated MC).

### Assay of β-hexosaminidase release

β-hexosaminidase (β-hex) secretion, a marker of MC degranulation was quantitated by fluorometric analysis of the hydrolysis of 4-methylumbelliferyl-N-acetyl-β-D-glucosaminide (Sigma Chemical Co.) as previously described [46]. The percentage of β-hex released into the supernatant was calculated by the following formula: [S/(S+P)]×100, where S and P are the β-hex contents of supernatant and cell pellet.

### Statistical Analysis

All experiments were performed at least three times. Data were analyzed using GraphPad Prism (version 5) and presented as mean ± SEM. p<0.05 was considered significant. Details of the statistical analyses used are indicated in figure legends.

## Results

### Expression of PGD_2_ receptors in human MC

**Figure 1:**
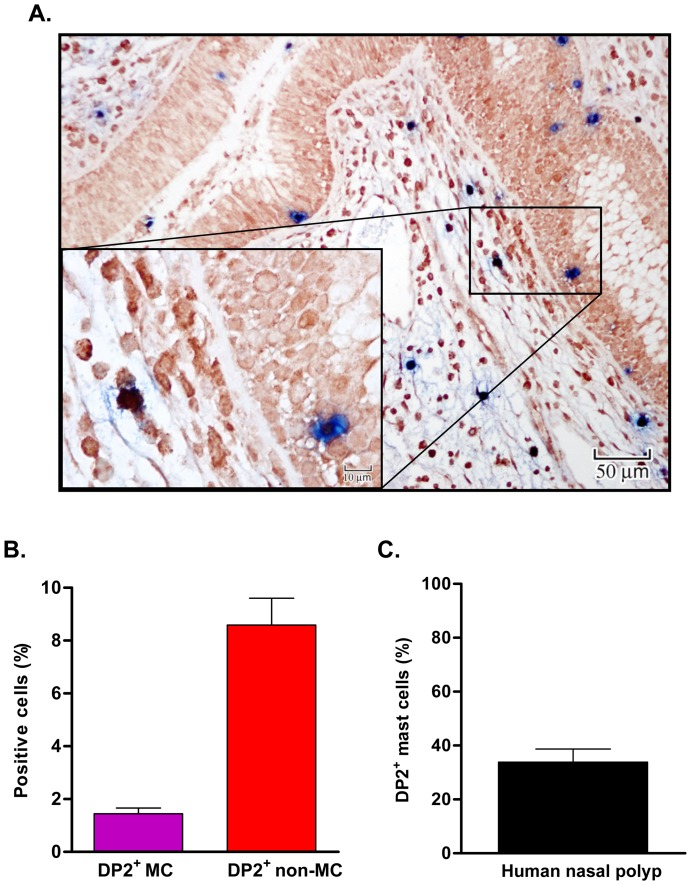
Immunohistochemical staining of DP2 in human nasal polyp mast cells. (A) Tissue sections from nasal polyps (n = 15) were double stained with rabbit anti-human DP2 and mouse anti-human MC tryptase antibodies or isotype matched control antibodies. DP2 staining is shown in dark red and MC tryptase is shown in blue. Insert shows the cellular staining with examples of single- (white triangle for DP2 single^+^, black triangle for tryptase single^+^) and double-positive cells (open arrow). (B) Percentage of DP2^+^ MC and non MC from total nucleated non epithelial cells (C) Percentage of DP2 positive MC among tryptase^+^ MC. The percentage of DP2 positive MC among MC was calculated by [number of double positive cells/(number of double positive cells + number of tryptase single positive cells)]×100.

Despite increasing evidence for DP2, also known as CRTh2, expression and function in various cell types (eg., human Th2 cells, eosinophils, basophils, dendritic cells) [27], [40], [41], little is known about expression and function of DP2 in MC [5]. We first examined expression of DP2 in MC *in situ* in human nasal polyp tissue. We previously reported that tryptase-positive MC were the dominant (> 99%) MC phenotype in human nasal polyp tissue (Fig 1A, black triangle in insert) [39]. As shown in Fig. 1A, DP2 positive cells in the nasal polyps included tryptase positive MC (double positive; white arrow in insert) and non MC (DP2 single positive; white triangle in insert). Using semiquantitative morphometric analyses of total nucleated cells below the epithelium, 1.5±0.2% were DP2 positive MC (double positive), and 9.2±0.8% were DP2 positive non-MC (DP2 single positive) (Fig 1B). In the nasal polyps, 33.9±4.8% of tryptase^+^ MC expressed DP2 (Fig 1C).

**Figure 2:**
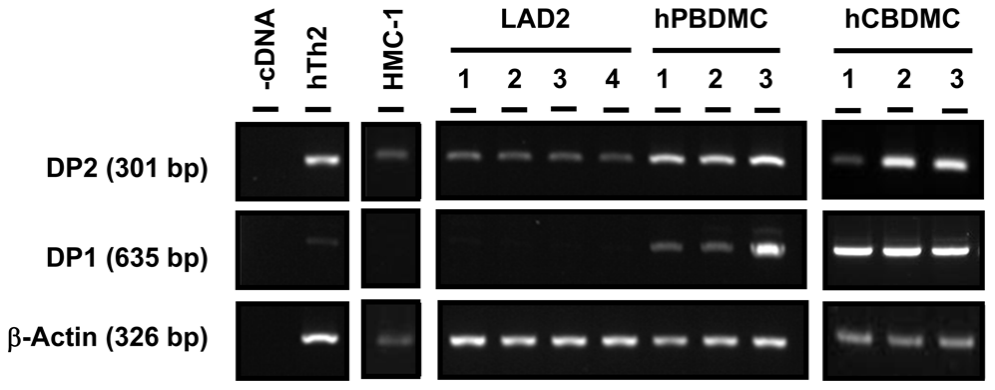
mRNA expression of PGD_2_ receptors in human mast cells. Expression of DP2 and DP1 mRNA in human MC lines (HMC-1 and LAD2) and primary cultured MC [peripheral blood-derived MC (hPBDMC) and cord blood-derived MC (hCBDMC)]. Three different cultures were shown for hPBDMC and hCBDMC. Human DP2^+^/CD4^+^ T cells cultured in Th2-polarizing conditions were used for a positive control of DP2 and dH_2_O instead of cDNA was used as a negative control.

**Figure 3:**
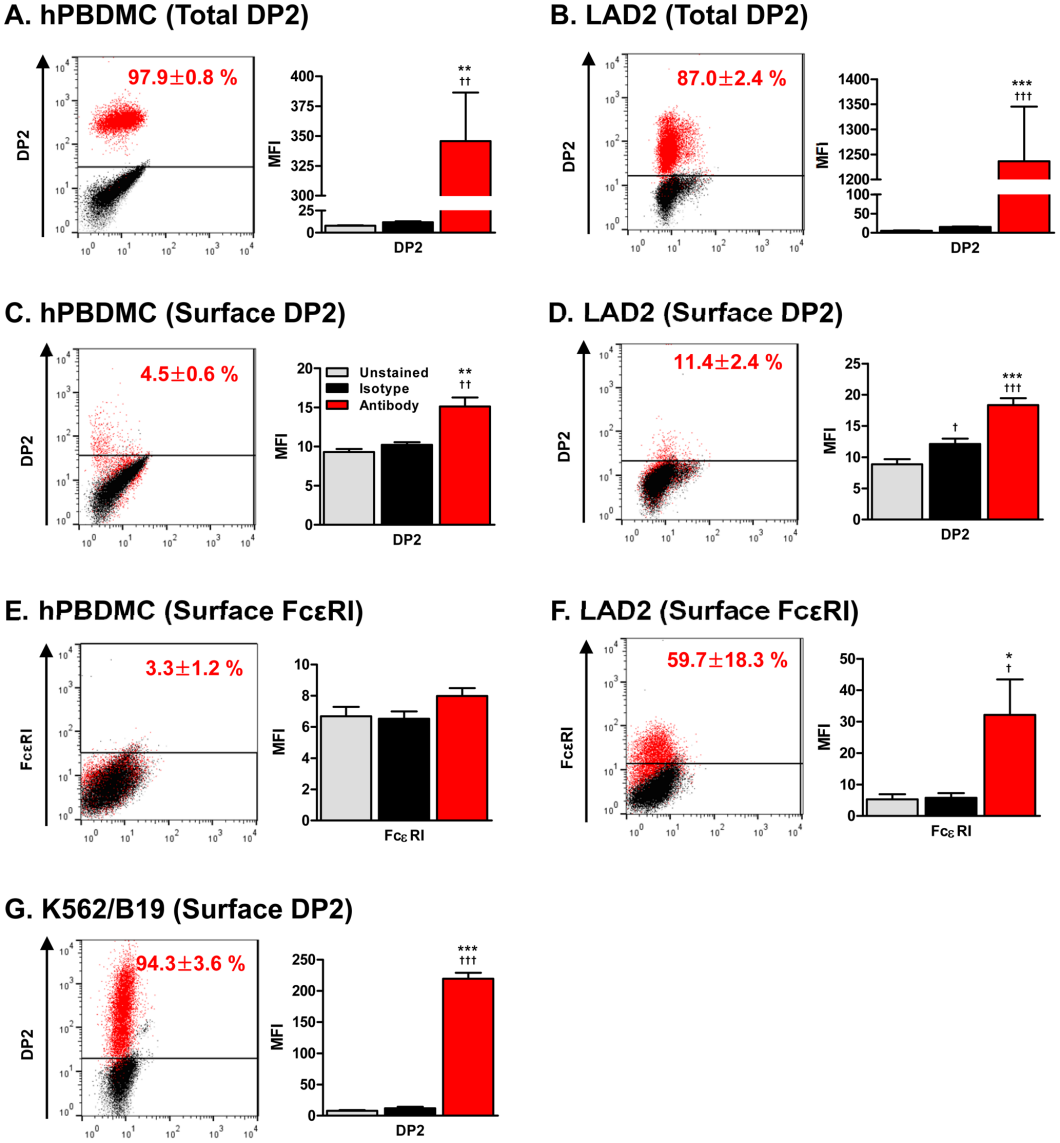
Flow cytometry analysis of DP2 and FcεRI expression on human mast cells. Expression of DP2 and FcεRI on hPBDMC and LAD2 were examined by flow cytometry. A representative result of dot plot (left) and MFI (Mean Fluorescent Intensity, right) from five to eight independent experiments calculated using WinMDI ver.2.9 software (mean±SEM) are shown. (A) Total expression of DP2 in hPBDMC (*n* = 8). (B) Total expression of DP2 in LAD2 (*n* = 5). (C) Surface expression of DP2 in hPBDMC (*n* = 8). (D) Surface expression of DP2 on LAD2 (*n* = 5). (E) Surface expression of FcεRI on hPBDMC (*n* = 15–21). (F) Surface expression of FcεRI (*n* = 5) on LAD2. (G) Surface expression of DP2 on DP2 transfectant, K562/B19 (*n* = 3) was examined as a control. In dot plot, the percentage of positive cells was shown inside, and gray, black and red dots represent unstained, stained with isotype control and specific antibody, respectively. ^†^p<0.05, ^††^p<0.01, ^†††^p<0.001 compared with unstained; **p<0.01, ***p<0.001 compared with isotype control by repeated measures ANOVA followed by the Tukey post-test.

We next examined expression of DP1 and DP2 in various human MC cultures. Two human MC lines, HMC-1 and LAD2, and two *in vitro* differentiated primary human MC, peripheral blood-derived MC (hPBDMC) and cord blood-derived MC (hCBDMC) expressed DP2 mRNA (Fig 2). The level of DP2 mRNA was higher in primary human MC than human MC lines. DP1 mRNA was also detected in human Th2 cells and in primary cultured human MC but not in human MC lines. In flow cytometry analysis, DP2 protein was detected by immunostaining after permeabilization in 97.9±0.8% and 87.0±2.4% of hPBDMC and LAD2, respectively (Fig 3A, B). However, surface expression of DP2 was observed only in 4.5±0.6% of hPBDMC and 11.4±2.4% of LAD2 (Fig 3C, D), and similar results were obtained using an independently generated rat anti-human DP2 antibody (IgG_2a_, clone BM16, Miltenyi Biotec) (Fig S1). Although DP2 expression on MC surface was low, it was comparable to FcεRI (3.3±1.2%, Fig 3E) on hPBDMC, a level that is functional in IgE-mediated mediator secretion (Fig 7B).

These results show that DP2 is expressed by human MC, and provided a rationale to examine the function of DP2 in human MC.

### DP2 agonist-induced cytosolic Ca^2+^ flux in human MC; sensitive to pertussis toxin but not to DP2 antagonists

**Figure 4:**
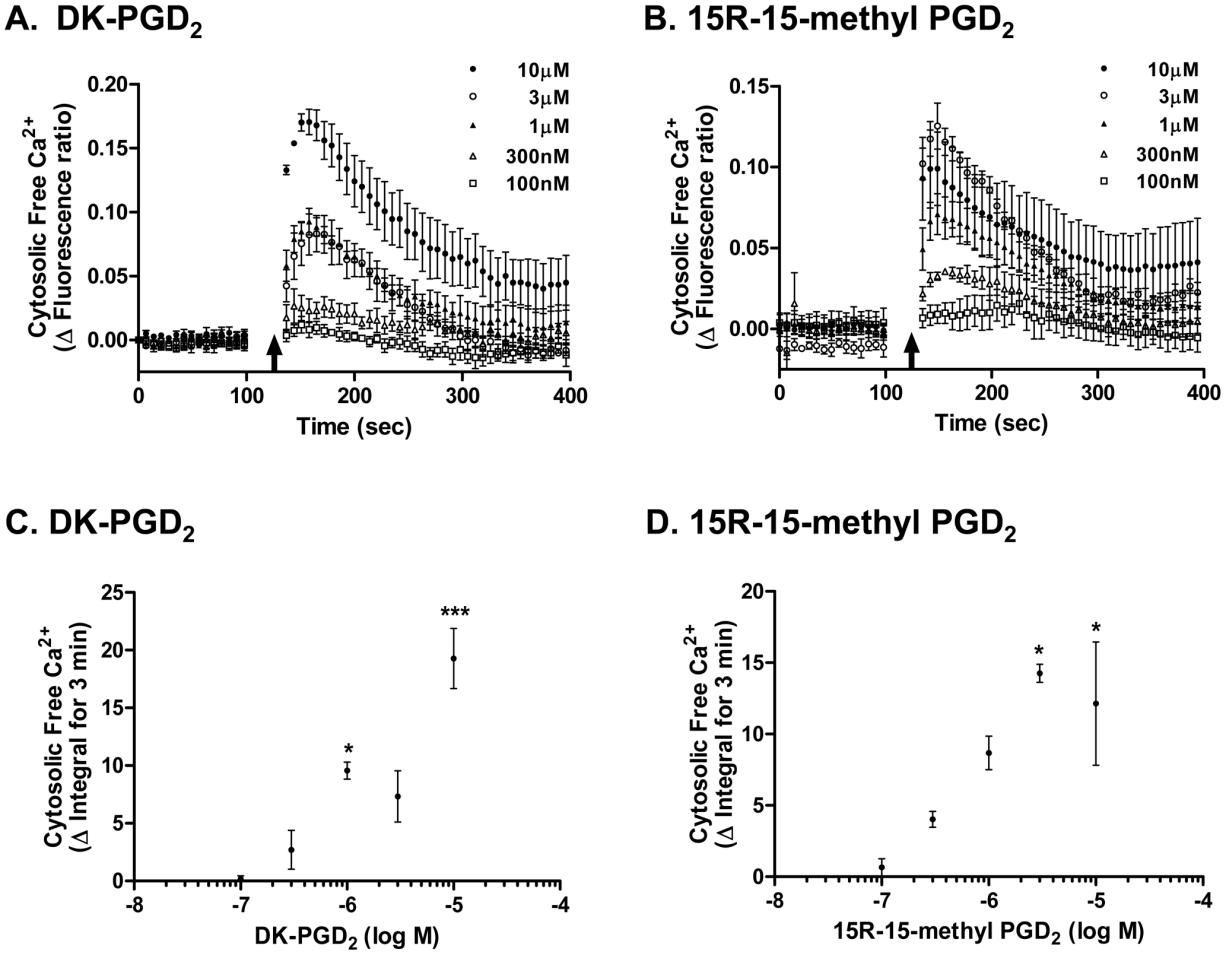
DP2 agonist-induced Ca^2+^ flux in human mast cells. After measuring baseline fluorescence of Fluo-4 AM loaded MC (1.25×10^5^ cells in 50 µL/well), (A, C) DK-PGD_2_ or (B, D) 15R-15-methyl PGD_2_ was given to the MC and intracellular Ca^2+^ flux was assessed by measuring fluorescence change. (A, B) Cytosolic free Ca^2+^ changes induced by DP2 agonists are presented as ΔFluorescence ratio (fluorescence ratio of agonist treatment – fluorescence ratio of sham treatment), where fluorescence ratio is fluorescence unit at each time point/baseline fluorescence unit. Arrow indicates the time when agonist was given. (C, D) Cytosolic free Ca^2+^ changes induced by DP2 agonist treatment are presented as ΔIntegral for 3 min from ΔFluorescent ratio curves shown in A and B. Results are expressed as mean ± SEM for three separate experiments. *p<0.05, **p<0.01 compared with 100 nM agonist treatment by repeated measures ANOVA followed by the Bonferroni post-test.

**Figure 5:**
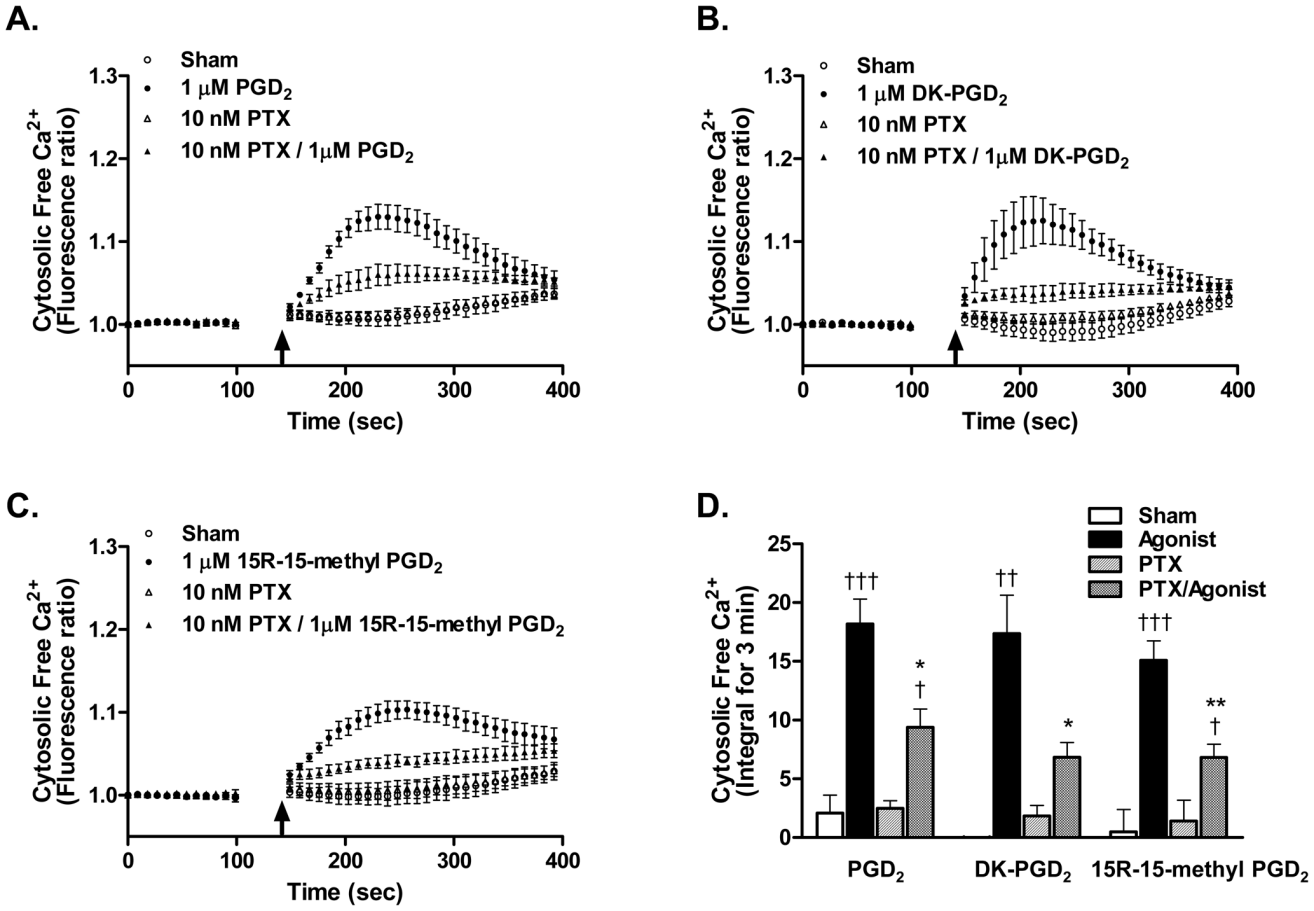
Pertussis toxin abolished DP2 agonist-induced Ca^2+^ flux in human mast cells. LAD2 were pretreated with 10 nM pertussis toxin (PTX) for 2 h then Fluo-4 AM was loaded. After measuring baseline fluorescence of Fluo-4 AM loaded MC (1.25×10^5^ cells in 50 µL/well), (A) 1 µM PGD_2_, (B) 1 µM DK-PGD_2_ or (C) 1 µM 15R-15-methyl PGD_2_ was added and intracellular Ca^2+^ flux was assessed by measuring fluorescence change. (A–C) Cytosolic free Ca^2+^ changes by DP2 agonists were presented as Fluorescence ratio (fluorescence unit at each time point/baseline fluorescence unit). Arrow indicates the time when agonist was given. (D) Cytosolic free Ca^2+^ changes in A–C are presented as integral for 3 min. Results are expressed as mean ± SEM for three separate experiments. ^†^p<0.05; ^††^p<0.01; ^†††^p<0.001 compared with each sham treatment (sham *vs* agonist, PTX *vs* PTX/agonist), *p<0.05; **p<0.01 compared with each agonist treatment (agonist *vs* PTX/agonist) by repeated measures ANOVA followed by the Tukey post-test.

To determine whether DP2 in MC is functional, cytosolic Ca^2+^ flux known to be downstream of DP2 signaling was assessed after treatment with DP2 agonists. Selective DP2 agonists, both physiologic and synthetic, DK-PGD_2_ (Fig 4A, C) and 15R-15-methyl PGD_2_ (Fig 4B, D) [47] respectively, induced a dose-dependent (100 nM to 10 µM) cytosolic Ca^2+^ flux in LAD2 cells. As DP2 receptors are coupled to Gα_i_ proteins for their signaling [20], we tested if the Ca^2+^ flux in MC induced by DP2 agonists was Gα_i_ mediated. LAD2 cells were pretreated with 10 nM pertussis toxin (PTX), which prevents G_i_ complex from interacting with receptors by ribosylation of Gα_i_ [48], [49]. As shown in Fig. 5, DP2 agonist-induced cytosolic Ca^2+^ flux was significantly diminished by PTX pretreatment. When cytosolic free Ca^2+^ was calculated by integral for 3 min, 10 nM PTX pretreatment inhibited 48.9±3.1% (p<0.05), 58.9±7.9% (p<0.05), and 55.1±5.2% (p<0.01) of 1 µM PGD_2_-, DK-PGD_2_-, and 15R-15methyl PGD_2_-induced cytosolic Ca^2+^ flux, respectively (Fig 5D). PTX treatment did not affect either cell viability, as reported previously [50], [51], or G_i_-independent Ca^2+^ flux induced by thapsigargin (Fig S2).

**Figure 6:**
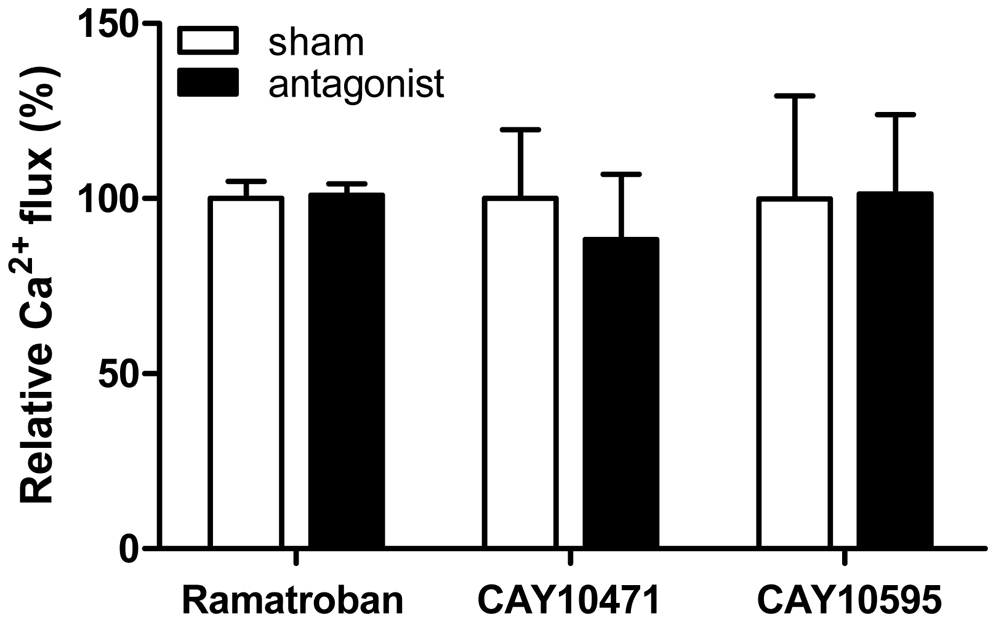
DP2 antagonists did not abolish DP2 agonist-induced intracellular Ca^2+^ flux. After measuring baseline fluorescence of Fluo-4 AM loaded LAD2, a DP2 selective antagonist (1 µM CAY10471 or 100 nM CAY10595) or DP2/TP dual antagonist (1 µM ramatroban) was added. After 5 min, 1 µM 15R-15-methyl PGD_2_ was added and intracellular Ca^2+^ flux was assessed by measuring fluorescence change. Relative Ca^2+^ flux was calculated from ΔIntegral for 3 min after addition of 15R-15-methyl PGD_2_, where sham treatment instead of antagonist considered as 100%. Results are expressed as mean ± SEM for three (ramatroban and CAY10471) and five (CAY10595) separate experiments. There was no statistical difference between sham and antagonist treatment. Note: Higher concentrations of each antagonist could not be used as they caused Ca^2+^ flux by themselves.

We next examined if this agonist-induced Ca^2+^ flux could be inhibited by DP2 selective antagonists. When MC were pretreated with either DP2 antagonists (1 µM CAY10471 or 100 nM CAY10595) or DP2/TP dual antagonist (1 µM ramatroban) for 5 min, there was no significant effect on DP2 agonist-induced Ca^2+^ flux in human MC (Fig 6).

These results suggest that intracellular Ca^2+^ flux by DP2 agonists occurs through a PTX-sensitive signaling pathway, but it cannot be unequivocally established that it is DP2-dependent.

### Effect of DP2 agonist, 15R-15 methyl PGD_2_ on mediator release of human MC

**Figure 7:**
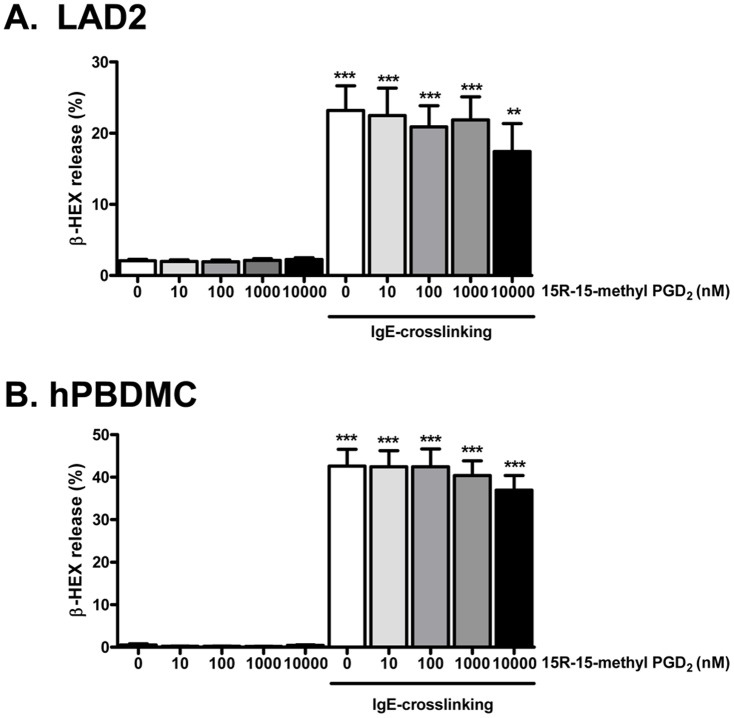
No effects of DP2 agonist on human mast cell degranulation induced by IgE-crosslinking. LAD2 or hPBDMC were sensitized with 100 ng/mL biotinylated human IgE overnight. Cells were washed and resuspended (2×10^5^ cells/200 µL) in HEPES-Tyrode's buffer (HTB), and stimulated with 100 ng/mL streptavidin in the presence or absence of indicated dose of 15R-15-methyl PGD_2_ for 30 min. The cells were centrifuged, and the percent release of β-HEX into the supernatant was calculated. β-HEX release (%) are expressed as mean ± SEM for 8–9 separate experiments of LAD2 (A), and 8–10 separate experiments of hPBDMC (B) with five different hPBDMC cultures. **p<0.01, ***p<0.001 compared with sham (0 nM 15R-15-methyl PGD_2_ without IgE cross-linking), and no statistical significant difference was found between 15R-15-methyl PGD_2_ treatment group by one-way ANOVA followed by the Tukey post-test.

Because DP2 activation induces degranulation of basophils [23] and eosinophils [21], we examined if a DP2 agonist could affect human MC degranulation. The DP2 selective agonist, 15R-15methyl PGD_2_ alone or in combination with IgE-crosslinking did not significantly alter degranulation (β-hex release) of LAD2 (Fig 7A) and hPBDMC (Fig 7B). Moreover, the DP2 selective agonist did not affect PGD_2_ and LTC_4_ release after IgE-crosslinking (Fig S3). Since PGD_2_ has been shown to induce Th2 cytokines from Th2 cells, we also examined IL-5 and IL-13 levels, but they were undetectable in both LAD2 and hPBDMC by DP2 agonist or IgE-crosslinking in the presence or absence of DP2 agonist (Table S1).

### Intracellular expression of DP2 in human MC

**Figure 8:**
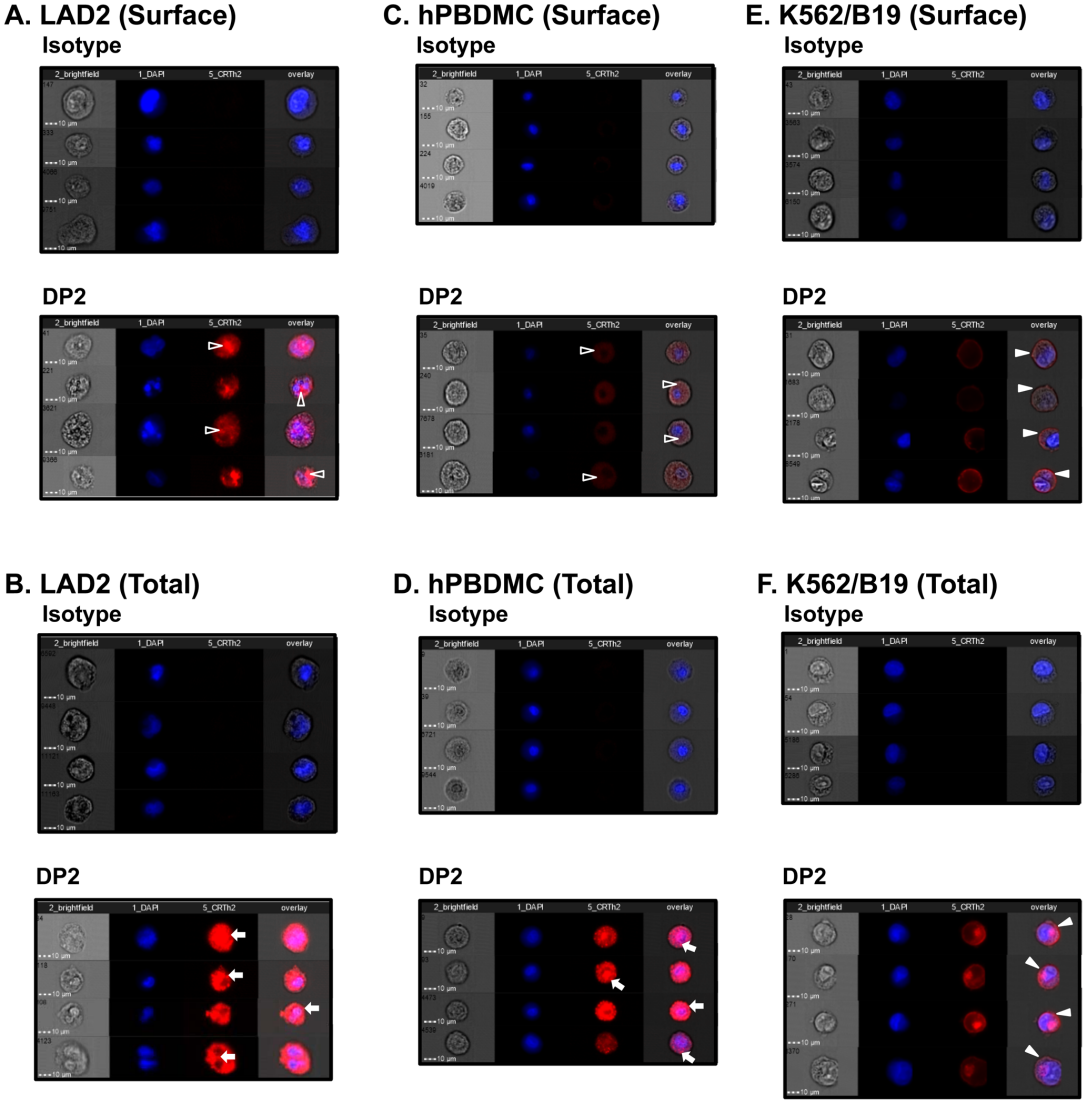
Single cell analysis of DP2 expression on human mast cells with ImageStream. Expression of DP2 on LAD2 and hPBDMC were examined with ImageStream after staining live cells for surface expression (A, C) or with fixed and permeabilized cells for total expression (B, D). (A, C) After surface staining, DP2 signals were detected from inside MC (open triangle) rather than on the surface. (B, D) Intracellular punctate staining for DP2 (arrow) was observed in fixed and permeabilized MC before staining. (E, F) K562/B19 (DP2 transfectant) was used as a control for surface expression of DP2 (closed triangle). Representative images of cells stained with isotype matched control Ab (left) and DP2 positive cells (right) from three independent experiments are shown.

Since DP2 selective antagonists failed to inhibit DP2 agonist induced Ca^2+^ flux (Fig 6) and DP2 selective agonists did not affect MC degranulation (Fig 7) or release of other mediators that we tested (Fig S3, Table S1), it is unclear whether the effect, although significantly inhibited by PTX (Fig 5), is actually due to DP2 activation. Moreover, although we confirmed that our methodology detects surface expression on the DP2 transfectant (Fig 3G), few MC (Fig 3C, D) showed surface expression of DP2, despite a high proportion of the MC expressing intracellular DP2. Thus, we examined MC expression of DP2 in further detail *via* imaging flow cytometry, a technique which merges fluorescence microscopy with flow cytometry allowing for robust quantitation of population-level morphological features based on single cell images [45]. In concordance with the conventional flow cytometry result shown in Fig 3, we detected extracellular staining for DP2 on 15.8±5.6% of LAD2 and 2.6±1.0% of PBDMC, and intracellular staining in 94.7±1.0% and 78.9±13.3% of permeabilized LAD2 and PBDMC, respectively. Surprisingly, however, by analyzing DP2^+^ cell images, we established that positive signals detected after surface staining were from inside MC (Fig 8A, C, open triangles), rather than on the surface. The ImageStream data also revealed that punctate staining for DP2 was observed in both LAD2 and PBDMC (Fig 8B, D, arrows) compared to DP2 transfected cells where DP2 was observed primarily on the cell surface (Fig 8E, F, closed triangle). Collectively, these data indicate that the majority of DP2 staining in human MC is found inside the cell.

### Effect of constitutive PGD_2_ on DP2 surface expression

**Figure 9:**
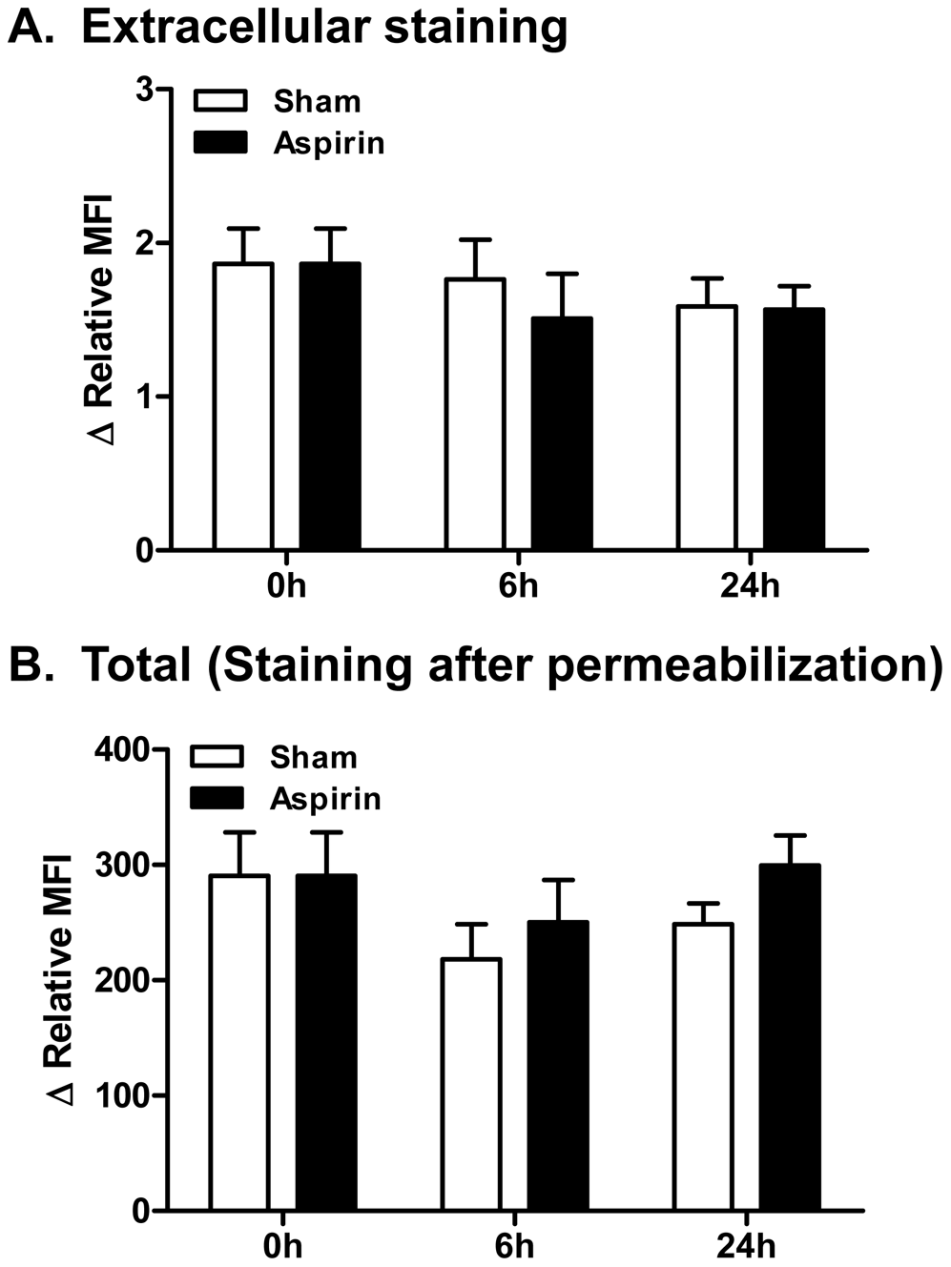
Effect of intrinsic PGD_2_ on DP2 expression in human mast cells. LAD2 were incubated for indicated time periods in the presence or absence of 10 µg/mL aspirin and then any change of surface and total DP2 expression was examined by flow cytometry. ΔRelative MFI [Relative mean fluorescent intensity (MFI) from stained cells with DP2 Ab - Relative MFI from stained cells with isotype Ab], where relative MFI is [(MFI from stained cells with Ab - MFI from unstained cells)/MFI from unstained cells], from five independent experiments was calculated using WinMDI ver.2.9 software (mean ± SEM). No statistical significance difference was found between sham and aspirin treatment groups by Two-way ANOVA followed by the Bonferroni post-test.

Because we detected low surface expression of DP2 on human MC (Fig 3C, D and 8) and it has been shown that DP2 is internalized when it binds its ligand [52], [53], we hypothesized that constitutive PGD_2_ produced by SCF-induced MC activation in cultures [54] might provide an explanation for the low DP2 surface expression in MC. To determine whether expression of DP2 on the surface of MC might be enhanced by blocking constitutive PGD_2_ production, we incubated LAD2 with 10 µg/mL aspirin and then examined expression of DP2 by flow cytometry. However, as shown in Fig. 9, blocking constitutive PGD_2_ production did not affect surface expression of DP2. This result suggests that expression of DP2 inside of MC, rather than on the surface, is not a result of ongoing internalization due to constitutive PGD_2_ production in our cultures.

In an effort to stimulate surface expression of DP2 on MC, we also tried several other approaches, including treatment with IFNγ and/or TNF, both which increase surface DP2 expression in eosinophils [55], treatment with IL-4, which induce DP2 expression in T cells [56] and SCF depletion from culture media to prohibit SCF-mediated MC activation [54]. However, all were unsuccessful (Fig S4). Collectively, this suggests that surface expression of DP2 in human MC is regulated differently than in other cell types (eg., eosinophils and Th2 cells).

## Discussion

PGD_2_ is the predominant prostanoid produced by activated MC and plays important roles in regulation of allergic inflammation, host defense, and innate and adaptive immune responses. However, there is a lack of understanding regarding expression of DP2 and its functional significance in human MC. In this study, we showed for the first time that human MC express DP2 (Figs 1–3, 8 and 9). Interestingly, DP2 expression was almost exclusively intracellular in human cultured MC (Figs 3, 8 and 9). We were unable to induce surface expression using several approaches including culture MC in surface DP2 increasing condition in eosinophils [55] or blocking constitutive PGD_2_ production (Fig 9). This suggests that surface expression of DP2 in human MC is regulated differently than in other cell types (eg., eosinophils).

Dose dependent cytosolic Ca^2+^ flux was detected in human MC after treatment with DP2 selective agonists (DK-PGD_2_ and 15R-15-methyl PGD_2_) in a range of 100 nM – 10 µM (Fig 4) and this was significantly depressed by PTX (Fig 5). However, human MC required significantly higher doses (1–10 µM) of DP2 agonists to induce Ca^2+^ flux than eosinophils [57]–[59] or Th2 cells (100 nM) [20], [60]. Moreover, DP2 selective antagonists did not inhibit DP2 agonist-induced Ca^2+^ flux in human MC, although we used higher dose than their IC_50_ shown previously in different experimental system [61]–[63]. However, we could not test higher doses of antagonists than shown in Fig 6, because these doses caused Ca^2+^ flux by themselves. Given these results, it is not clear whether the intracellular Ca^2+^ flux is DP2 dependent or not. The predominant intracellular expression of DP2 rather than on the cell surface of human MC may explain the need for a high dose of agonist to induce the Ca^2+^ signal through intracellular DP2. PGD_2_ and DK-PGD_2_ have poor permeability of the cell membrane, although there is no information about PGD_2_ uptake in human MC and there is no direct evidence that human MC express the prostaglandin transporter on the cell surface [64]–[67]. The synthetic DP2 agonist (15R-15-methyl PGD_2_) and DP2 antagonists have not been characterized in terms of their cell permeability. Further study of the permeability of DP2 agonists and antagonists will help to clarify DP2 dependency of intracellular Ca^2+^ flux shown in this study.

Although DP2 signaling can induce Th2 cytokine production [19], degranulation [21], [23] and leukotriene production [29], [68] in other immune cells, we could not detect any of these responses in human MC stimulated with DP2 agonists. The lack of effect of DP2 agonists on MC mediator release suggest that the role of intracellular DP2 in MC is different than its role on the surface of other immune cells such as Th2 cells [19], eosinophils [21], [29], [68], and basophils [23]. Our results about the high dose of DP2 agonist required for Ca^2+^ mobilization and the lack of effect of DP2 agonists on MC degranulation and cytokine release are consistent with those shown in mouse MC [31].

With advances in analytical methods that enable imaging at subcellular resolution, there is increasing evidence of intracellular expression of GPCRs, and this reveals distinct functions and signal transduction mechanisms inside cells as compared with the plasma membrane location [69], [70]. For example, β2-adrenoreceptor, a prototypical GPCR, signaling occurs intracellularly in the endosome, as well as in the plasma membrane [69], and more recently, Binda *et al* [70] showed that the intracellular expression of DP1 is associated with intracrine/autocrine signaling of DP1 mediated by ERK1/2 in the perinuclear region. Although further study is needed to define the intracellular location of DP2 in human MC, we observed punctate staining of DP2 in human MC (Fig 8), typical of endosomal or granular expression. Therefore, intracellular DP2 in human MC may play an unknown role in MC function, distinct from the functions of cell surface DP2. Given the recent finding of a role for lipocalin-type prostaglandin D synthase (L-PGDS) and heat shock proteins in trafficking of DP1 to the cell membrane [70], cell surface trafficking of DP2 may also be regulated by other proteins. Although L-PGDS did not mediate trafficking of other GPCRs, including DP2 [70], they did not test hematopoietic PGDS (H-PGDS), the relevant PGDS in MC. The study of trafficking mechanisms of DP2 will help determine the role of different cellular locations of this receptor in different cell types.

In this study we showed intracellular expression of DP2 in human MC. Although DP2 agonists induced Ca^2+^ flux and this was abolished with PTX, DP2 antagonists failed to inhibit Ca^2+^ flux induced by agonist. Therefore DP2 dependency or not of agonist-induced Ca^2+^ flux needs further clarification. DP2 agonists neither induced nor augmented β-HEX release, or eicosanoids (PGD_2_ and LTC_4_) and cytokine (IL-5 and IL-13) production in the presence or absence of FcεRI crosslinking. This may be related with its location inside the cell rather than on the surface, and permeability of DP2 agonists and antagonists. Despite all our efforts, we were unable to induce surface expression or translocation of DP2 from inside to the surface. Further study is needed to understand trafficking and functional significance of DP2 in human MC. Understanding its functional significance in human MC and their responses may help inform development and application of DP2 antagonists for therapeutic intervention.

## Supporting Information

Figure S1 **Flow cytometry analysis of DP2 expression on human mast cells.** Expression of DP2 on hPBDMC and LAD2 were examined by flow cytometry using rat anti-human DP2 antibody (IgG2a, clone BM16). Percentage of positive cells (left) and MFI (Mean Fluorescent Intensity, right) from three to four independent experiments calculated using WinMDI ver.2.9 software (mean±SEM) are shown. (A) Surface expression of DP2 in hPBDMC (*n* = 3). (B) Surface expression of DP2 on LAD2 (*n* = 4). (C) Total expression of DP2 on LAD2 (*n* = 1, triplicate). *p <0.05, **p <0.01 compared with isotype control by one-tailed paired t-test.
(TIF)

Figure S2 **PTX pretreatment did not affect thapsigargin induced Ca^2+^ flux and viability of human mast cells.** A–C. LAD2 were pretreated with 10 nM pertussis toxin (PTX) for 2 h then Fluo-4 AM was loaded. After measuring baseline fluorescence of Fluo-4 AM loaded MC (1.25×10^5^ cells in 50 µL/well), 10 µM 15R-15-methyl PGD_2_ (A) or 1 µM thapsigargin (Sigma) (B) was added and intracellular Ca^2+^ flux was assessed by measuring fluorescence change. Cytosolic free Ca^2+^ changes by stimulation were presented as Fluorescence ratio (fluorescence unit at each time point/baseline fluorescence unit). Arrow indicates the time when stimulus was given. Cytosolic free Ca^2+^ changes in A and B are presented as integral for 3 min (C). Results are expressed as mean ± SEM for three separate experiments with duplication. ^††^p<0.01; ^†††^p<0.001 compared with each sham treatment (sham *vs* stimulus, PTX *vs* PTX/stimulus), **p<0.01 compared with each stimulus treatment; NS, not significant (stimulus *vs* PTX/stimulus) by one-way ANOVA followed by the Tukey post-test. D. Cell viability after PTX treatment was measured with WST-1 according to manufacturer's instruction (Roche Applied science, 68298 Mannheim, Germany). LAD2 (5×10^4^ cells in 100 µL/well) were treated with 10 nM PTX for 2 h and then 10 µL of WST-1 was added to the well. After 2 h incubation, absorbance at 440 nm and 690 nm were measured and results are expressed as mean ± SEM of background subtracted A_440_–A_690_ values from triplicated experiment. NS, not significant by one-tailed t-test.
(TIF)

Figure S3 **DP2 agonist did not affect FcεRI-mediated PGD_2_ and LTC_4_ production of human mast cell.** hPBDMC or LAD2 were sensitized with 100 ng/mL biotinylated human IgE overnight. Cells were washed and stimulated with 100 ng/mL streptavidin in the presence or absence of indicated dose of 15R-15-methyl PGD_2_ or PGD_2_ for 30 min. The cells were centrifuged, and the release of PGD_2_ or LTC_4_ into the supernatant was measured by ELISA (Cayman Chemical). A. Effect of 15R-15-methyl PGD_2_ on FcεRI-mediated PGD_2_ release from hPBDMC (*n* = 1). Note that 15R-15-methyl PGD_2_ did not cross-react with PGD_2_ ELISA. PGD_2_ detected in the presence of 1000 ng/mL 15R-15-methyl PGD_2_ was 0.8 ng/ml. B. Effect of 15R-15-methyl PGD_2_ on FcεRI-mediated LTC_4_ release from hPBDMC. **p<0.01 compared with unstimulated control, but not significant (NS) in the presence or absence of 15R-15-methyl PGD_2_ by repeated measures ANOVA followed by the Tukey post-test (*n* = 4). C. Effect of 15R-15-methyl PGD_2_ on FcεRI-mediated LTC_4_ release from LAD2 (*n* = 1). D. Effect of PGD_2_ on FcεRI-mediated LTC_4_ release from hPBDMC (*n* = 2).
(TIF)

Figure S4 **IL-4, SCF starvation, and IFNγ and/or TNF did not affect surface expression level of DP2 on human mast cells.** hPBDMC (A, *n* = 2) and LAD2 (B, *n* = 3–4) were cultured in the presence or absence of 100 ng/ml rhIL-4 for 7 days then expression of DP2 on their surface were examined by flow cytometry. FcεRI expression was examined as internal control for IL-4 effect. C. Expression of DP2 on the surface of LAD2 was examined after 1 day starvation of SCF. CD117 expression was examined as internal control for SCF starvation (*n* = 1). D. DP2 expression was examined after 1 day of LAD2 culture in the presence or absence of IFNγ and/or TNF (*n* = 2). Relative MFI was calculated by MFI of stained cells with antibody/MFI of stained cells with isotype control.
(TIF)

Table S1 **DP2 agonist did not induce IL-5 and IL-13 production from human mast cells in the presence or absence of FcεRI-crosslinking.**
(DOCX)

## References

[1] Marshall JS (2004) Mast-cell responses to pathogens. Nat Rev Immunol 4: 787–799. 15459670 10.1038/nri1460

[2] Galli SJ, Nakae S, Tsai M (2005) Mast cells in the development of adaptive immune responses. Nat Immunol 6: 135–142. 15662442 10.1038/ni1158

[3] Galli SJ, Kalesnikoff J, Grimbaldeston MA, Piliponsky AM, Williams CM, et al (2005) Mast cells as "tunable" effector and immunoregulatory cells: recent advances. Annu Rev Immunol 23: 749–786. 15771585 10.1146/annurev.immunol.21.120601.141025

[4] Moon TC, St Laurent CD, Morris KE, Marcet C, Yoshimura T, et al (2010) Advances in mast cell biology: new understanding of heterogeneity and function. Mucosal Immunol 3: 111–128. 20043008 10.1038/mi.2009.136

[5] Lewis RA, Soter NA, Diamond PT, Austen KF, Oates JA, et al (1982) Prostaglandin D2 generation after activation of rat and human mast cells with anti-IgE. J Immunol 129: 1627–1631. 6809826

[6] Boyce JA (2005) Eicosanoid mediators of mast cells: receptors, regulation of synthesis, and pathobiologic implications. Chem Immunol Allergy 87: 59–79. 16107763 10.1159/000087571

[7] Peters-Golden M, Canetti C, Mancuso P, Coffey MJ (2005) Leukotrienes: underappreciated mediators of innate immune responses. J Immunol 174: 589–594. 15634873 10.4049/jimmunol.174.2.589

[8] Joo M, Sadikot RT (2012) PGD synthase and PGD2 in immune response. Mediators Inflamm 2012: 503128. 22791937 10.1155/2012/503128 PMC3389719

[9] Obata T, Nagakura T, Kanbe M, Masaki T, Maekawa K, et al (1996) IgE-anti-IgE-induced prostaglandin D2 release from cultured human mast cells. Biochem Biophys Res Commun 225: 1015–1020. 8780726 10.1006/bbrc.1996.1287

[10] Urade Y, Ujihara M, Horiguchi Y, Ikai K, Hayaishi O (1989) The major source of endogenous prostaglandin D2 production is likely antigen-presenting cells. Localization of glutathione-requiring prostaglandin D synthetase in histiocytes, dendritic, and Kupffer cells in various rat tissues. J Immunol 143: 2982–2989. 2509561

[11] Matsuoka T, Hirata M, Tanaka H, Takahashi Y, Murata T, et al (2000) Prostaglandin D2 as a mediator of allergic asthma. Science 287: 2013–2017. 10720327 10.1126/science.287.5460.2013

[12] Pettipher R, Hansel TT, Armer R (2007) Antagonism of the prostaglandin D2 receptors DP1 and CRTH2 as an approach to treat allergic diseases. Nat Rev Drug Discov 6: 313–325. 17396136 10.1038/nrd2266

[13] Bochenek G, Nizankowska E, Gielicz A, Swierczynska M, Szczeklik A (2004) Plasma 9alpha,11beta-PGF2, a PGD2 metabolite, as a sensitive marker of mast cell activation by allergen in bronchial asthma. Thorax 59: 459–464. 15170023 10.1136/thx.2003.013573 PMC1747024

[14] Johnston SL, Freezer NJ, Ritter W, O'Toole S, Howarth PH (1995) Prostaglandin D2-induced bronchoconstriction is mediated only in part by the thromboxane prostanoid receptor. Eur Respir J 8: 411–415. 7789486 10.1183/09031936.95.08030411

[15] Underwood DC, Muccitelli RM, Luttmann MA, Hay DW, Torphy TJ, et al (1994) Differential antagonism of airway contractile responses to prostaglandin (PG)D2 and 9 alpha, 11 beta-PGF2 by atropine, SK&F 88046 and SQ 29,548 in the guinea pig. J Pharmacol Exp Ther 268: 304–310. 8301572

[16] Beasley R, Hovel C, Mani R, Robinson C, Varley J, et al (1988) Comparative vascular effects of histamine, prostaglandin (PG) D2 and its metabolite 9 alpha,11 beta-PGF2 in human skin. Clin Allergy 18: 619–627. 3242978 10.1111/j.1365-2222.1988.tb02914.x

[17] Fuller RW, Dixon CM, Dollery CT, Barnes PJ (1986) Prostaglandin D2 potentiates airway responsiveness to histamine and methacholine. Am Rev Respir Dis 133: 252–254. 3511806 10.1164/arrd.1986.133.2.252

[18] Gosset P, Bureau F, Angeli V, Pichavant M, Faveeuw C, et al (2003) Prostaglandin D2 affects the maturation of human monocyte-derived dendritic cells: consequence on the polarization of naive Th cells. J Immunol 170: 4943–4952. 12734337 10.4049/jimmunol.170.10.4943

[19] Xue L, Gyles SL, Wettey FR, Gazi L, Townsend E, et al (2005) Prostaglandin D2 causes preferential induction of proinflammatory Th2 cytokine production through an action on chemoattractant receptor-like molecule expressed on Th2 cells. J Immunol 175: 6531–6536. 16272307 10.4049/jimmunol.175.10.6531

[20] Hirai H, Tanaka K, Yoshie O, Ogawa K, Kenmotsu K, et al (2001) Prostaglandin D2 selectively induces chemotaxis in T helper type 2 cells, eosinophils, and basophils via seven-transmembrane receptor CRTH2. J Exp Med 193: 255–261. 11208866 10.1084/jem.193.2.255 PMC2193345

[21] Gervais FG, Cruz RP, Chateauneuf A, Gale S, Sawyer N, et al (2001) Selective modulation of chemokinesis, degranulation, and apoptosis in eosinophils through the PGD2 receptors CRTH2 and DP. J Allergy Clin Immunol 108: 982–988. 11742277 10.1067/mai.2001.119919

[22] Mackay GA, Stewart AG (2011) R2D(2) for C(4) Eo: an 'alliance' of PGD(2) receptors is required for LTC(4) production by human eosinophils. Br J Pharmacol 162: 1671–1673. 21426314 10.1111/j.1476-5381.2011.01236.x PMC3081112

[23] Yoshimura-Uchiyama C, Iikura M, Yamaguchi M, Nagase H, Ishii A, et al (2004) Differential modulation of human basophil functions through prostaglandin D2 receptors DP and chemoattractant receptor-homologous molecule expressed on Th2 cells/DP2. Clin Exp Allergy 34: 1283–1290. 15298571 10.1111/j.1365-2222.2004.02027.x

[24] Tajima T, Murata T, Aritake K, Urade Y, Hirai H, et al (2008) Lipopolysaccharide induces macrophage migration via prostaglandin D(2) and prostaglandin E(2). J Pharmacol Exp Ther 326: 493–501. 18492946 10.1124/jpet.108.137992

[25] Taketomi Y, Ueno N, Kojima T, Sato H, Murase R, et al (2013) Mast cell maturation is driven via a group III phospholipase A2-prostaglandin D2-DP1 receptor paracrine axis. Nat Immunol 14: 554–563. 23624557 10.1038/ni.2586 PMC4065307

[26] Nagata K, Hirai H (2003) The second PGD(2) receptor CRTH2: structure, properties, and functions in leukocytes. Prostaglandins Leukot Essent Fatty Acids 69: 169–177. 12895600 10.1016/s0952-3278(03)00078-4

[27] Kostenis E, Ulven T (2006) Emerging roles of DP and CRTH2 in allergic inflammation. Trends Mol Med 12: 148–158. 16545607 10.1016/j.molmed.2006.02.005

[28] Abe H, Takeshita T, Nagata K, Arita T, Endo Y, et al (1999) Molecular cloning, chromosome mapping and characterization of the mouse CRTH2 gene, a putative member of the leukocyte chemoattractant receptor family. Gene 227: 71–77. 9931443 10.1016/s0378-1119(98)00599-x

[29] Mesquita-Santos FP, Bakker-Abreu I, Luna-Gomes T, Bozza PT, Diaz BL, et al (2011) Co-operative signalling through DP(1) and DP(2) prostanoid receptors is required to enhance leukotriene C(4) synthesis induced by prostaglandin D(2) in eosinophils. Br J Pharmacol 162: 1674–1685. 20973774 10.1111/j.1476-5381.2010.01086.x PMC3081113

[30] Shirasaki H, Kikuchi M, Kanaizumi E, Himi T (2009) Accumulation of CRTH2-positive leukocytes in human allergic nasal mucosa. Ann Allergy Asthma Immunol 102: 110–115. 19230460 10.1016/S1081-1206(10)60239-6

[31] Boehme SA, Franz-Bacon K, Chen EP, Ly TW, Kawakami Y, et al (2009) Murine bone marrow-derived mast cells express chemoattractant receptor-homologous molecule expressed on T-helper class 2 cells (CRTh2). Int Immunol. 10.1093/intimm/dxp031 19346259

[32] Hirai H, Abe H, Tanaka K, Takatsu K, Sugamura K, et al (2003) Gene structure and functional properties of mouse CRTH2, a prostaglandin D2 receptor. Biochem Biophys Res Commun 307: 797–802. 12878180 10.1016/s0006-291x(03)01266-x

[33] Butterfield JH, Weiler D, Dewald G, Gleich GJ (1988) Establishment of an immature mast cell line from a patient with mast cell leukemia. Leuk Res 12: 345–355. 3131594 10.1016/0145-2126(88)90050-1

[34] Kirshenbaum AS, Akin C, Wu Y, Rottem M, Goff JP, et al (2003) Characterization of novel stem cell factor responsive human mast cell lines LAD 1 and 2 established from a patient with mast cell sarcoma/leukemia; activation following aggregation of FcepsilonRI or FcgammaRI. Leuk Res 27: 677–682. 12801524 10.1016/s0145-2126(02)00343-0

[35] Sekar Y, Moon TC, Slupsky CM, Befus AD (2010) Protein tyrosine nitration of aldolase in mast cells: A plausible pathway in nitric oxide-mediated regulation of mast cell function. J Immunol 185: 578–587. 20511553 10.4049/jimmunol.0902720

[36] Kirshenbaum AS, Goff JP, Semere T, Foster B, Scott LM, et al (1999) Demonstration that human mast cells arise from a progenitor cell population that is CD34(+), c-kit(+), and expresses aminopeptidase N (CD13). Blood 94: 2333–2342. 10498605

[37] Marcet CW, St Laurent CD, Moon TC, Singh N, Befus AD (2013) Limited replication of influenza A virus in human mast cells. Immunol Res 56: 32–43. 23055084 10.1007/s12026-012-8377-4

[38] Moon TC, Lee E, Baek SH, Murakami M, Kudo I, et al (2003) Degranulation and cytokine expression in human cord blood-derived mast cells cultured in serum-free medium with recombinant human stem cell factor. Mol Cells 16: 154–160. 14651255

[39] Yoshimura T, Moon TC, St Laurent CD, Puttagunta L, Chung K, et al (2012) Expression of nitric oxide synthases in leukocytes in nasal polyps. Ann Allergy Asthma Immunol 108: 172–177. 22374200 10.1016/j.anai.2011.12.013

[40] Nagata K, Hirai H, Tanaka K, Ogawa K, Aso T, et al (1999) CRTH2, an orphan receptor of T-helper-2-cells, is expressed on basophils and eosinophils and responds to mast cell-derived factor(s). FEBS Lett 459: 195–199. 10518017 10.1016/s0014-5793(99)01251-x

[41] Nagata K, Tanaka K, Ogawa K, Kemmotsu K, Imai T, et al (1999) Selective expression of a novel surface molecule by human Th2 cells in vivo. J Immunol 162: 1278–1286. 9973380

[42] Clarke DL, Belvisi MG, Smith SJ, Hardaker E, Yacoub MH, et al (2005) Prostanoid receptor expression by human airway smooth muscle cells and regulation of the secretion of granulocyte colony-stimulating factor. Am J Physiol Lung Cell Mol Physiol 288: L238–250. 15640521 10.1152/ajplung.00313.2004

[43] Ulanova M, Puttagunta L, Marcet-Palacios M, Duszyk M, Steinhoff U, et al (2005) Syk tyrosine kinase participates in beta1-integrin signaling and inflammatory responses in airway epithelial cells. Am J Physiol Lung Cell Mol Physiol 288: L497–507. 15557085 10.1152/ajplung.00246.2004

[44] Campos Alberto E, Maclean E, Davidson C, Palikhe NS, Storie J, et al (2012) The single nucleotide polymorphism CRTh2 rs533116 is associated with allergic asthma and increased expression of CRTh2. Allergy 67: 1357–1364. 22947041 10.1111/all.12003

[45] Zuba-Surma EK, Kucia M, Abdel-Latif A, Lillard JW Jr, Ratajczak MZ (2007) The ImageStream System: a key step to a new era in imaging. Folia Histochem Cytobiol 45: 279–290. 18165167

[46] Moon TC, Yoshimura T, Parsons T, Befus AD (2012) Microenvironmental regulation of inducible nitric oxide synthase expression and nitric oxide production in mouse bone marrow-derived mast cells. J Leukoc Biol 91: 581–590. 22262798 10.1189/jlb.0811436

[47] Monneret G, Cossette C, Gravel S, Rokach J, Powell WS (2003) 15R-methyl-prostaglandin D2 is a potent and selective CRTH2/DP2 receptor agonist in human eosinophils. J Pharmacol Exp Ther 304: 349–355. 12490611 10.1124/jpet.102.042937

[48] Katada T, Ui M (1982) Direct modification of the membrane adenylate cyclase system by islet-activating protein due to ADP-ribosylation of a membrane protein. Proc Natl Acad Sci U S A 79: 3129–3133. 6954463 10.1073/pnas.79.10.3129 PMC346367

[49] Katada T (2012) The inhibitory G protein G(i) identified as pertussis toxin-catalyzed ADP-ribosylation. Biol Pharm Bull 35: 2103–2111. 23207763 10.1248/bpb.b212024

[50] Befus AD, Mowat C, Gilchrist M, Hu J, Solomon S, et al (1999) Neutrophil defensins induce histamine secretion from mast cells: mechanisms of action. J Immunol 163: 947–953. 10395691

[51] Grigat J, Soruri A, Forssmann U, Riggert J, Zwirner J (2007) Chemoattraction of macrophages, T lymphocytes, and mast cells is evolutionarily conserved within the human alpha-defensin family. J Immunol 179: 3958–3965. 17785833 10.4049/jimmunol.179.6.3958

[52] Roy SJ, Parent A, Gallant MA, de Brum-Fernandes AJ, Stankova J, et al (2010) Characterization of C-terminal tail determinants involved in CRTH2 receptor trafficking: identification of a recycling motif. Eur J Pharmacol 630: 10–18. 20035740 10.1016/j.ejphar.2009.12.022

[53] Hamada K, Yamada Y, Kamada Y, Ueki S, Yamaguchi K, et al (2004) Prostaglandin D2 and interleukin-5 reduce CRTH2 surface expression on human eosinophils. Allergology International 53: 179–184.

[54] Columbo M, Horowitz EM, Botana LM, MacGlashan DW Jr, Bochner BS, et al (1992) The human recombinant c-kit receptor ligand, rhSCF, induces mediator release from human cutaneous mast cells and enhances IgE-dependent mediator release from both skin mast cells and peripheral blood basophils. J Immunol 149: 599–608. 1378071

[55] El-Shazly AE, Moonen V, Mawet M, Begon D, Henket M, et al (2011) IFN-gamma and TNF-alpha potentiate prostaglandin D2-induced human eosinophil chemotaxis through up-regulation of CRTH2 surface receptor. Int Immunopharmacol 11: 1864–1870. 21835268 10.1016/j.intimp.2011.07.017

[56] Huber JP, Ramos HJ, Gill MA, Farrar JD (2010) Cutting edge: Type I IFN reverses human Th2 commitment and stability by suppressing GATA3. J Immunol 185: 813–817. 20554961 10.4049/jimmunol.1000469 PMC2927323

[57] Bohm E, Sturm GJ, Weiglhofer I, Sandig H, Shichijo M, et al (2004) 11-Dehydro-thromboxane B2, a stable thromboxane metabolite, is a full agonist of chemoattractant receptor-homologous molecule expressed on TH2 cells (CRTH2) in human eosinophils and basophils. J Biol Chem 279: 7663–7670. 14668348 10.1074/jbc.M310270200

[58] Schuligoi R, Sedej M, Waldhoer M, Vukoja A, Sturm EM, et al (2009) Prostaglandin H2 induces the migration of human eosinophils through the chemoattractant receptor homologous molecule of Th2 cells, CRTH2. J Leukoc Biol 85: 136–145. 18835884 10.1189/jlb.0608387

[59] Chiba T, Ueki S, Ito W, Kato H, Kamada R, et al (2011) The opposing role of two prostaglandin D2 receptors, DP and CRTH2, in human eosinophil migration. Ann Allergy Asthma Immunol 106: 511–517. 21624751 10.1016/j.anai.2011.01.027

[60] Hirai H, Tanaka K, Takano S, Ichimasa M, Nakamura M, et al (2002) Cutting edge: agonistic effect of indomethacin on a prostaglandin D2 receptor, CRTH2. J Immunol 168: 981–985. 11801628 10.4049/jimmunol.168.3.981

[61] Ulven T, Kostenis E (2005) Minor structural modifications convert the dual TP/CRTH2 antagonist ramatroban into a highly selective and potent CRTH2 antagonist. J Med Chem 48: 897–900. 15715457 10.1021/jm049036i

[62] Crosignani S, Page P, Missotten M, Colovray V, Cleva C, et al (2008) Discovery of a new class of potent, selective, and orally bioavailable CRTH2 (DP2) receptor antagonists for the treatment of allergic inflammatory diseases. J Med Chem 51: 2227–2243. 18318469 10.1021/jm701383e

[63] Sugimoto H, Shichijo M, Okano M, Bacon KB (2005) CRTH2-specific binding characteristics of [3H]ramatroban and its effects on PGD2-, 15-deoxy-Delta12, 14-PGJ2- and indomethacin-induced agonist responses. Eur J Pharmacol 524: 30–37. 16256979 10.1016/j.ejphar.2005.09.005

[64] Lu R, Kanai N, Bao Y, Schuster VL (1996) Cloning, in vitro expression, and tissue distribution of a human prostaglandin transporter cDNA(hPGT). J Clin Invest 98: 1142–1149. 8787677 10.1172/JCI118897 PMC507536

[65] Chan BS, Satriano JA, Pucci M, Schuster VL (1998) Mechanism of prostaglandin E2 transport across the plasma membrane of HeLa cells and Xenopus oocytes expressing the prostaglandin transporter "PGT". J Biol Chem 273: 6689–6697. 9506966 10.1074/jbc.273.12.6689

[66] Schuster VL (1998) Molecular mechanisms of prostaglandin transport. Annu Rev Physiol 60: 221–242. 9558462 10.1146/annurev.physiol.60.1.221

[67] Bao Y, Pucci ML, Chan BS, Lu R, Ito S, et al (2002) Prostaglandin transporter PGT is expressed in cell types that synthesize and release prostanoids. Am J Physiol Renal Physiol 282: F1103–1110. 11997327 10.1152/ajprenal.00152.2001

[68] Mesquita-Santos FP, Vieira-de-Abreu A, Calheiros AS, Figueiredo IH, Castro-Faria-Neto HC, et al (2006) Cutting edge: prostaglandin D2 enhances leukotriene C4 synthesis by eosinophils during allergic inflammation: synergistic in vivo role of endogenous eotaxin. J Immunol 176: 1326–1330. 16424158 10.4049/jimmunol.176.3.1326

[69] Irannejad R, Tomshine JC, Tomshine JR, Chevalier M, Mahoney JP, et al (2013) Conformational biosensors reveal GPCR signalling from endosomes. Nature 495: 534–538. 23515162 10.1038/nature12000 PMC3835555

[70] Binda C, Genier S, Cartier A, Larrivee JF, Stankova J, et al (2014) A G protein-coupled receptor and the intracellular synthase of its agonist functionally cooperate. J Cell Biol 204: 377–393. 24493589 10.1083/jcb.201304015 PMC3912537

