# Correction: Expression of DP2 (CRTh2), a Prostaglandin D_2_ Receptor, in Human Mast Cells

**DOI:** 10.1371/journal.pone.0116246

**Published:** 2014-12-17

**Authors:** The PLOS ONE Staff

There are errors in Figure 1(A) of the published article. Please see the correct Figure 1 here.

**Figure 1:**
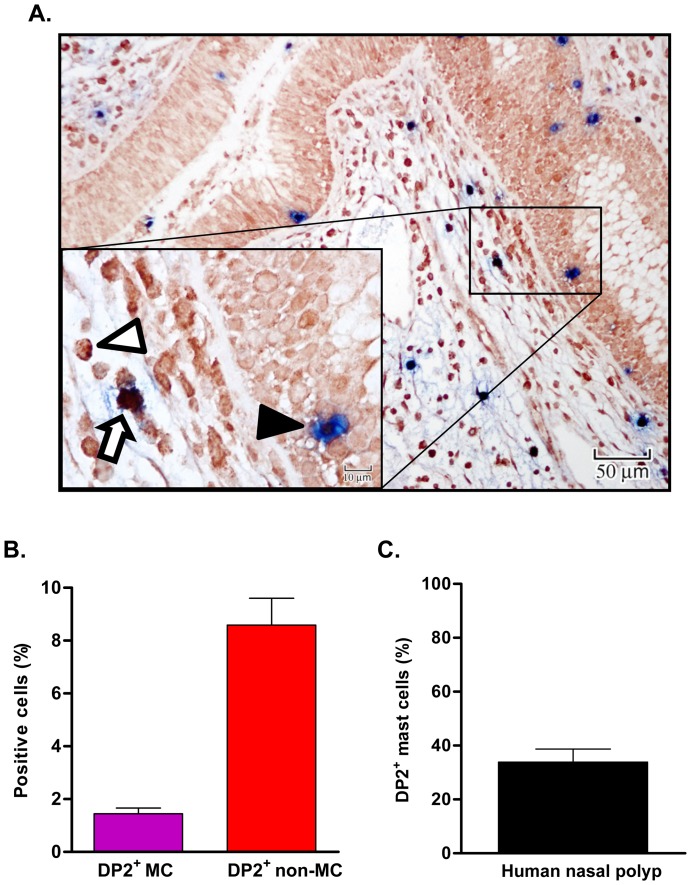
Immunohistochemical staining of DP2 in human nasal polyp mast cells. (A) Tissue sections from nasal polyps (n  =  15) were double stained with rabbit anti-human DP2 and mouse anti-human MC tryptase antibodies or isotype matched control antibodies. DP2 staining is shown in dark red and MC tryptase is shown in blue. Insert shows the cellular staining with examples of single- (white triangle for DP2 single^+^, black triangle for tryptase single^+^) and double-positive cells (open arrow). (B) Percentage of DP2^+^ MC and non MC from total nucleated non epithelial cells (C) Percentage of DP2 positive MC among tryptase^+^ MC. The percentage of DP2 positive MC among MC was calculated by [number of double positive cells/(number of double positive cells + number of tryptase single positive cells)]×100.

## References

[1] Moon TC, Campos-Alberto E, Yoshimura T, Bredo G, Rieger AM, et al (2014) Expression of DP2 (CRTh2), a Prostaglandin D_2_ Receptor, in Human Mast Cells. PLoS ONE 9(9): e108595 doi:10.1371/journal.pone.0108595 25268140 10.1371/journal.pone.0108595 PMC4182489

