# Supplementary figures and images for: Expression of DP2 (CRTh2), a Prostaglandin D2 Receptor, in Human Mast Cells
Source: PLoS One. 2014 Sep 30;9(9):e108595. doi: 10.1371/journal.pone.0108595 (PMC4182489; doi:10.1371/journal.pone.0108595)

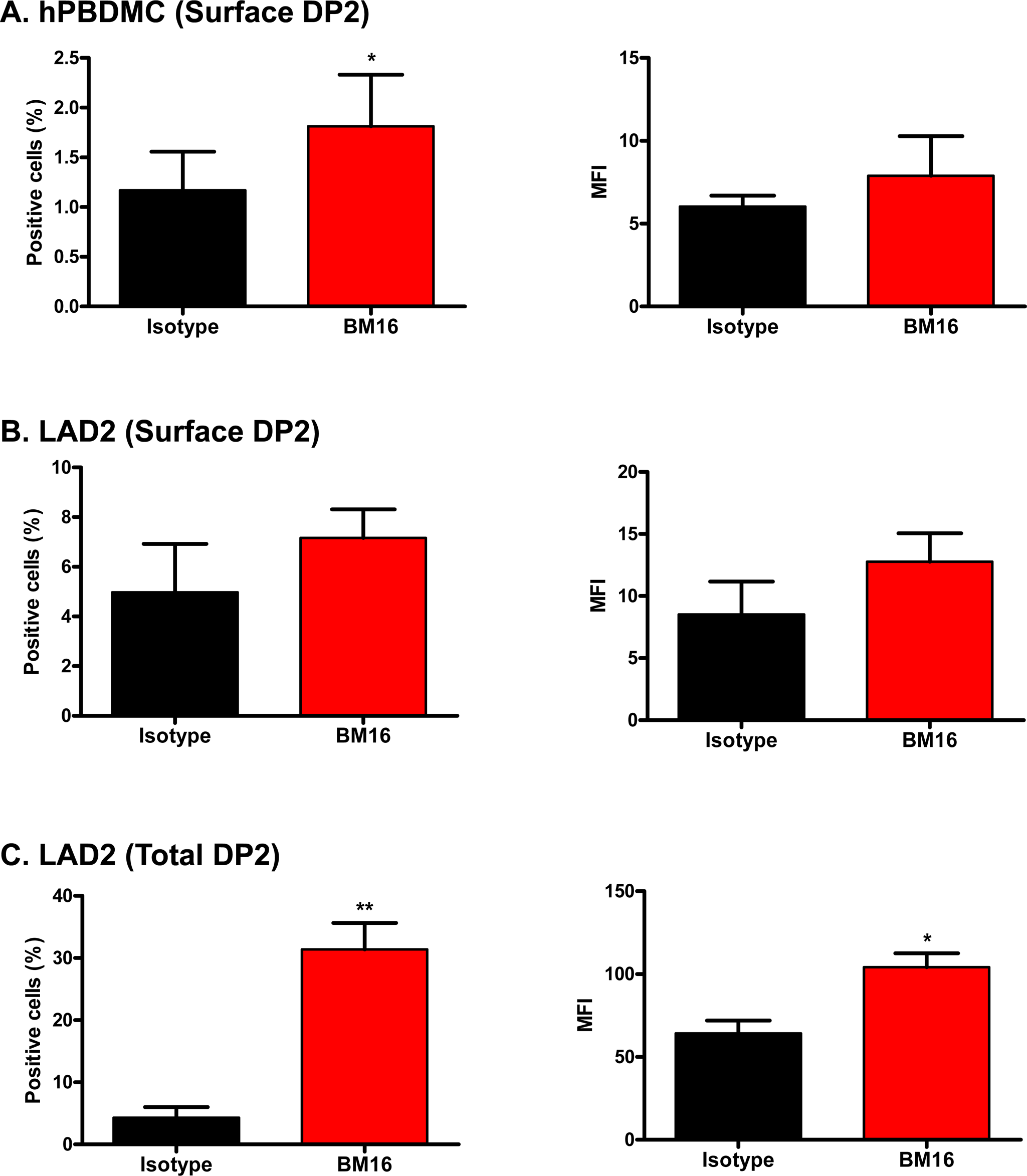

Supplement: Figure S1 — Flow cytometry analysis of DP2 expression on human mast cells. Expression of DP2 on hPBDMC and LAD2 were examined by flow cytometry using rat anti-human DP2 antibody (IgG2a, clone BM16). Percentage of positive cells (left) and MFI (Mean Fluorescent Intensity, right) from three to four independent experiments calculated using WinMDI ver.2.9 software (mean±SEM) are shown. (A) Surface expression of DP2 in hPBDMC (n = 3). (B) Surface expression of DP2 on LAD2 (n = 4). (C) Total expression of DP2 on LAD2 (n = 1, triplicate). *p <0.05, **p <0.01 compared with isotype control by one-tailed paired t-test. (TIF) [file pone.0108595.s001.tif]

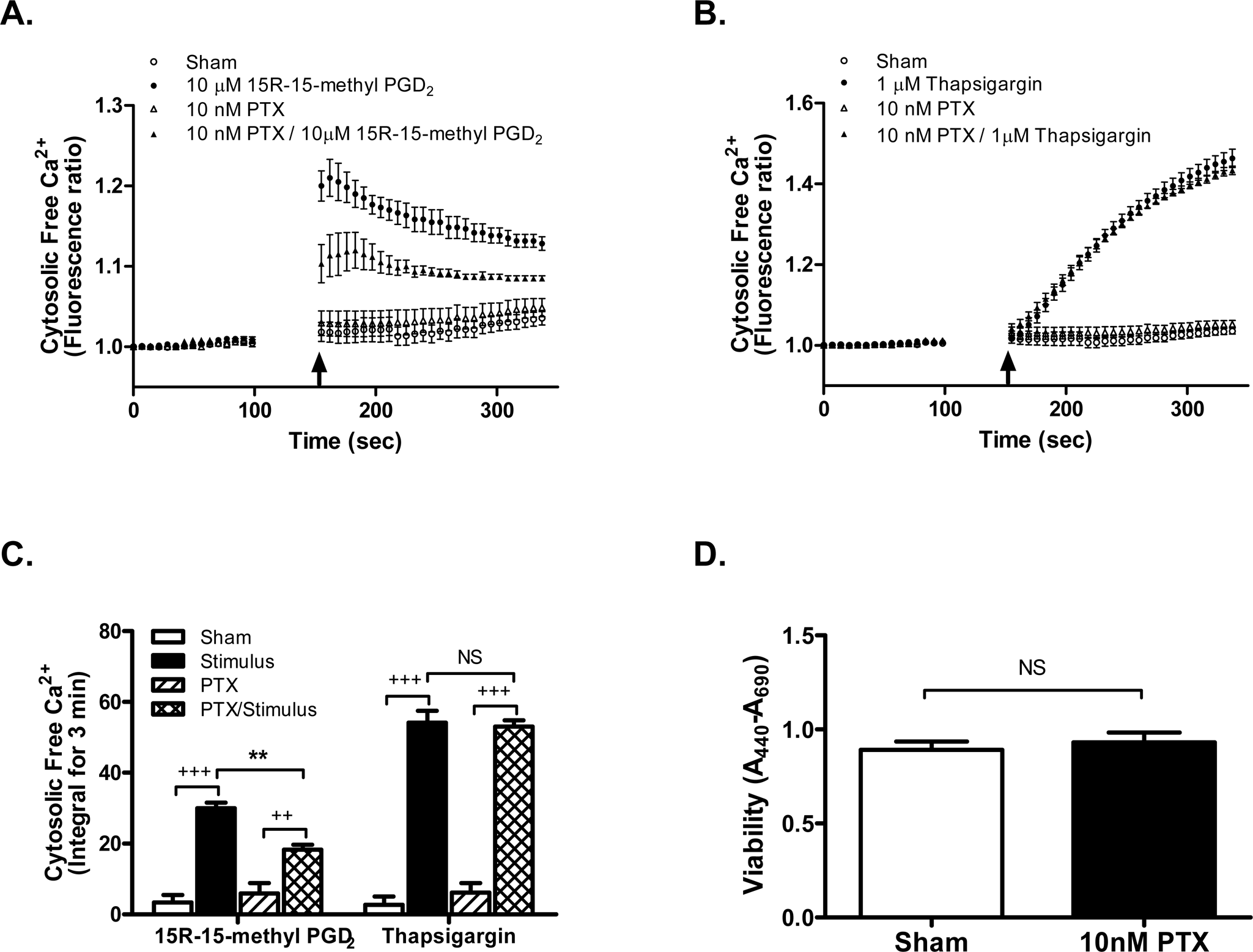

Supplement: Figure S2 — PTX pretreatment did not affect thapsigargin induced Ca2+ flux and viability of human mast cells. A–C. LAD2 were pretreated with 10 nM pertussis toxin (PTX) for 2 h then Fluo-4 AM was loaded. After measuring baseline fluorescence of Fluo-4 AM loaded MC (1.25×10^5^ cells in 50 µL/well), 10 µM 15R-15-methyl PGD2 (A) or 1 µM thapsigargin (Sigma) (B) was added and intracellular Ca2+ flux was assessed by measuring fluorescence change. Cytosolic free Ca2+ changes by stimulation were presented as Fluorescence ratio (fluorescence unit at each time point/baseline fluorescence unit). Arrow indicates the time when stimulus was given. Cytosolic free Ca2+ changes in A and B are presented as integral for 3 min (C). Results are expressed as mean ± SEM for three separate experiments with duplication. ††p<0.01; †††p<0.001 compared with each sham treatment (sham vs stimulus, PTX vs PTX/stimulus), **p<0.01 compared with each stimulus treatment; NS, not significant (stimulus vs PTX/stimulus) by one-way ANOVA followed by the Tukey post-test. D. Cell viability after PTX treatment was measured with WST-1 according to manufacturer's instruction (Roche Applied science, 68298 Mannheim, Germany). LAD2 (5×10^4^ cells in 100 µL/well) were treated with 10 nM PTX for 2 h and then 10 µL of WST-1 was added to the well. After 2 h incubation, absorbance at 440 nm and 690 nm were measured and results are expressed as mean ± SEM of background subtracted A440–A690 values from triplicated experiment. NS, not significant by one-tailed t-test. (TIF) [file pone.0108595.s002.tif]

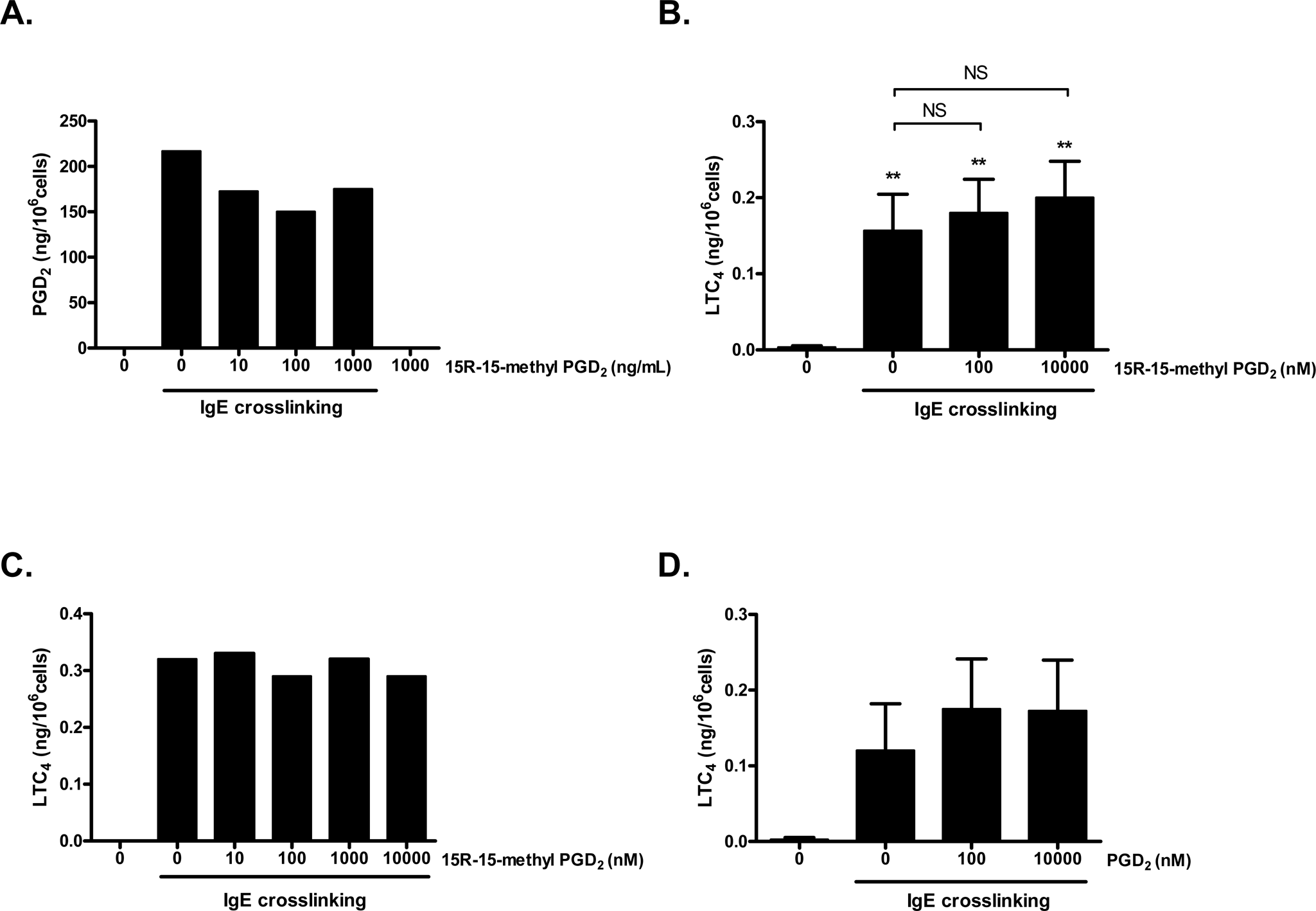

Supplement: Figure S3 — DP2 agonist did not affect FcεRI-mediated PGD2 and LTC4 production of human mast cell. hPBDMC or LAD2 were sensitized with 100 ng/mL biotinylated human IgE overnight. Cells were washed and stimulated with 100 ng/mL streptavidin in the presence or absence of indicated dose of 15R-15-methyl PGD2 or PGD2 for 30 min. The cells were centrifuged, and the release of PGD2 or LTC4 into the supernatant was measured by ELISA (Cayman Chemical). A. Effect of 15R-15-methyl PGD2 on FcεRI-mediated PGD2 release from hPBDMC (n = 1). Note that 15R-15-methyl PGD2 did not cross-react with PGD2 ELISA. PGD2 detected in the presence of 1000 ng/mL 15R-15-methyl PGD2 was 0.8 ng/ml. B. Effect of 15R-15-methyl PGD2 on FcεRI-mediated LTC4 release from hPBDMC. **p<0.01 compared with unstimulated control, but not significant (NS) in the presence or absence of 15R-15-methyl PGD2 by repeated measures ANOVA followed by the Tukey post-test (n = 4). C. Effect of 15R-15-methyl PGD2 on FcεRI-mediated LTC4 release from LAD2 (n = 1). D. Effect of PGD2 on FcεRI-mediated LTC4 release from hPBDMC (n = 2). (TIF) [file pone.0108595.s003.tif]

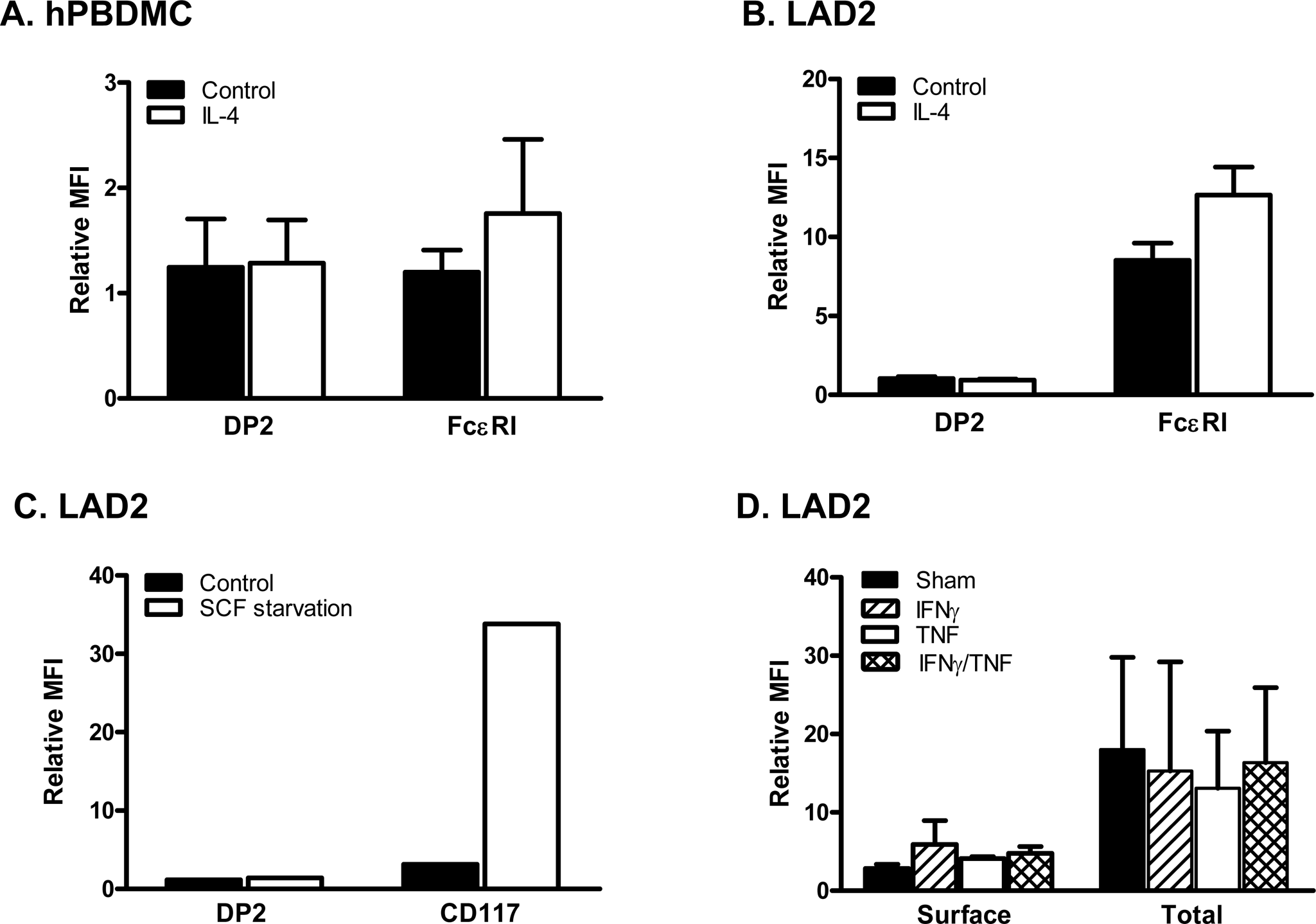

Supplement: Figure S4 — IL-4, SCF starvation, and IFNγ and/or TNF did not affect surface expression level of DP2 on human mast cells. hPBDMC (A, n = 2) and LAD2 (B, n = 3–4) were cultured in the presence or absence of 100 ng/ml rhIL-4 for 7 days then expression of DP2 on their surface were examined by flow cytometry. FcεRI expression was examined as internal control for IL-4 effect. C. Expression of DP2 on the surface of LAD2 was examined after 1 day starvation of SCF. CD117 expression was examined as internal control for SCF starvation (n = 1). D. DP2 expression was examined after 1 day of LAD2 culture in the presence or absence of IFNγ and/or TNF (n = 2). Relative MFI was calculated by MFI of stained cells with antibody/MFI of stained cells with isotype control. (TIF) [file pone.0108595.s004.tif]
