# Supplementary material for: Expression of DP2 (CRTh2), a Prostaglandin D2 Receptor, in Human Mast Cells
Source: PLoS One. 2014 Sep 30;9(9):e108595. doi: 10.1371/journal.pone.0108595 (PMC4182489; doi:10.1371/journal.pone.0108595)
Supplement: Table S1 — DP2 agonist did not induce IL-5 and IL-13 production from human mast cells in the presence or absence of FcεRI-crosslinking. (DOCX) [file pone.0108595.s005.docx]

**Supplemental Table 1.** DP2 agonist did not induce IL-5 and IL-13 production from human mast cells in the presence or absence of FcεRI-crosslinking

| FcεRI crosslinking | | - | + | - | - | - | - | + | + | + | + |
| --- | --- | --- | --- | --- | --- | --- | --- | --- | --- | --- | --- |
| 15R-15-methyl PGD_2_ (nM) | | 0 | 0 | 10 | 100 | 1000 | 10000 | 10 | 100 | 1000 | 10000 |
| hPBDMC | IL-5 | UDL^a^ | UDL | UDL | UDL | UDL | UDL | UDL | UDL | UDL | UDL |
|  | IL-13 | UDL | UDL | UDL | UDL | UDL | UDL | UDL | UDL | UDL | UDL |
| LAD2 | IL-5 | UDL | UDL | UDL | UDL | UDL | UDL | UDL | UDL | UDL | UDL |
|  | IL-13 | UDL | UDL | UDL | UDL | UDL | UDL | UDL | UDL | UDL | UDL |

hPBDMC or LAD2 were sensitized with 100 ng/mL biotinylated human IgE overnight. Cells were washed and stimulated with 100 ng/mL streptavidin in the presence or absence of indicated dose of 15R-15-methyl PGD_2_ for 24 h. The cells were centrifuged, and the release of IL-5 and IL-13 into the supernatant was measured by ELISA^b^.

^a^UDL: Under detection limit in two independent experiments, IL-5 and IL-13 ELISA detection limits are 1.08 pg/ml and 1.5 pg/ml, respectively.

^b^ELISA: IL-5 and IL-13 ELISA kit were purchased from R&D Systems and Diaclone (25020 Besançon cedex, France), respectively.
